# Tailoring optical nonlinearity and gamma-ray shielding in $\:{\mathbf{B}\mathbf{i}}_{2}{\mathbf{O}}_{3}$-modified borate–BCZT glasses

**DOI:** 10.1038/s41598-026-53588-z

**Published:** 2026-06-04

**Authors:** Esraa A. Gaber, S. A. Hussien, E. M Saad, Moukhtar A. Hassan

**Affiliations:** 1 Ferroelectric & Piezoelectric Lab, Physics Department, Faculty of Science, Qena University, Qena, 83523 Egypt; 2 Physics Department, Faculty of Science, Al-Azhar University, Nasr City, Cairo, E- 11884 Egypt

**Keywords:** Modified borate glasses, Band gap engineering, Nonlinear optical properties, Non-bridging oxygen (NBO), Gamma-ray attenuation., Chemistry, Materials science, Physics

## Abstract

This study investigates the structural, optical, nonlinear, and gamma-ray shielding properties of $\:{\mathrm{B}\mathrm{i}}_{2}{\mathrm{O}}_{3}$–$\:{\mathrm{B}}_{2}{\mathrm{O}}_{3}$–BCZT glasses with composition x$\:{\mathrm{B}\mathrm{i}}_{2}{\mathrm{O}}_{3}$ + (30–x) $\:{\mathrm{B}}_{2}{\mathrm{O}}_{3}$ + 70[($\:{\mathrm{B}\mathrm{a}}_{0.85}{\mathrm{C}\mathrm{a}}_{0.15}$)( $\:{\mathrm{T}\mathrm{i}}_{0.9}{\mathrm{Z}\mathrm{r}}_{0.1}$)$\:{\mathrm{O}}_{3}$] (x = 0–25 mol%) synthesized via the conventional melt-quenching technique. X-ray diffraction (XRD) confirmed the amorphous nature of all samples, while density increased from 3.622 to 5.788 g/cm³ (~ 60% enhancement) with increasing $\:{\mathrm{B}\mathrm{i}}_{2}{\mathrm{O}}_{3}$ content, accompanied by an increase in molar volume, indicating significant modification of the glass network. Fourier transform infrared (FTIR) analysis revealed enhanced Bi–O bond formation and an increase in non-bridging oxygen (NBO), confirming network depolymerization. Optical absorption analysis showed a decrease in the optical band gap from 3.615 to 2.839 eV, accompanied by an increase in Urbach energy, indicating enhanced structural disorder and the formation of localized states. The nonlinear optical parameters exhibited significant enhancement due to the high polarizability of Bi³⁺ ions. In addition, radiation shielding performance, evaluated using Phy-X/PSD in the energy range 15 keV–15 MeV, showed improved mass attenuation coefficient and effective atomic number, and a reduced half-value layer with increasing $\:{\mathrm{B}\mathrm{i}}_{2}{\mathrm{O}}_{3}$ content. This work presents a systematic, concept-driven approach to tailoring borate–BCZT glasses via controlled $\:{\mathrm{B}\mathrm{i}}_{2}{\mathrm{O}}_{3}$ incorporation, enabling stable glass formation even at low $\:{\mathrm{B}}_{2}{\mathrm{O}}_{3}$ former content (down to 5 mol%). More importantly, the study establishes a quantitative structure–property–function relationship linking $\:{\mathrm{B}\mathrm{i}}_{2}{\mathrm{O}}_{3}$-induced structural modifications with the simultaneous enhancement of the nonlinear optical response and gamma-ray shielding performance. Unlike previous studies that treat these properties separately, the present work demonstrates their integrated optimization within a single system, highlighting the potential of these glasses for advanced photonic and radiation protection applications.

## Introduction

Glass materials have attracted significant scientific interest owing to their diverse physical, chemical, and optical properties. They are widely employed for various technological uses, including photonics, optoelectronics, and radiation shielding, because of their high transparency, tunable composition, and chemical durability^1^. Glass is currently an essential and significant part of numerous industries, including building materials, communications, photovoltaics, optoelectronic devices, and automobiles. The practicality of glass has continued to improve, propelling technological innovation^2^.

Oxide glasses represent one of the most extensively studied and technologically important categories of amorphous materials due to their wide range of characteristics, which are very advantageous for many applications. These glasses are primarily composed of metal oxides, with the basic structural framework built from interconnected oxygen polyhedral^3^. Among amorphous materials, oxide glasses have attracted considerable attention because of their compositional flexibility, optical transparency, chemical durability, and ease of processing. In particular, borate-based glasses are regarded as promising host matrices owing to their low melting temperature, high transparency, good thermal stability, and strong ability to accommodate a wide range of network modifiers and intermediate oxides. Their structure is mainly derived from $\:{\mathrm{B}}_{2}{\mathrm{O}}_{3}$ and consists of $\:{\mathrm{B}\mathrm{O}}_{3}$ and $\:{\mathrm{B}\mathrm{O}}_{4}$ units whose relative distribution can be readily altered by compositional modification. This high structural adaptability makes borate glasses suitable for the design of materials with tailored physical and optical properties^2,4^. One of the most effective approaches for improving borate glass performance is the incorporation of heavy metal oxides such as $\:{\mathrm{B}\mathrm{i}}_{2}{\mathrm{O}}_{3}$. Because of its high atomic mass, large ionic radius, and strong electronic polarizability, $\:{\mathrm{B}\mathrm{i}}_{2}{\mathrm{O}}_{3}$ can significantly enhance density, refractive index, and photon interaction probability. At the structural level, $\:{\mathrm{B}\mathrm{i}}_{2}{\mathrm{O}}_{3}$ also acts as a network modifier, disrupting B–O–B linkages, promoting the formation of non-bridging oxygens, and altering the BO₃/BO₄ ratio within the borate network [5,^6^. These changes are highly relevant because they can simultaneously affect both the optical response and the shielding efficiency. Consequently, bismuth-borate glasses have been extensively explored for applications requiring wide optical transparency and high functional performance, including optical fibers, sensors, and transparent radiation shields^4^.

In parallel, the lead-free perovskite composition ($\:{\mathrm{B}\mathrm{a}}_{0.85}{\mathrm{C}\mathrm{a}}_{0.15}$)( $\:{\mathrm{T}\mathrm{i}}_{0.9}{\mathrm{Z}\mathrm{r}}_{0.1}$)$\:{\mathrm{O}}_{3}$ designated as (BCZT) has been widely recognized for its excellent dielectric, piezoelectric, and ferroelectric behavior in the ceramic state^7^. Its high dielectric constant, strong electromechanical response, and environmental compatibility make it a promising alternative to lead-based materials. However, the synthesis of BCZT ceramics generally requires high calcination and sintering temperatures (> 1350 °C), which complicates processing^8^. When BCZT-related constituents are introduced into a glass matrix, their role changes from long-range ferroelectric ordering to local structural and electronic modification. In such amorphous systems, Ti- and Zr-containing units can influence network connectivity, polarizability, localized electronic transitions, and optical absorption in the UV–Vis–NIR range^9,10^. Therefore, BCZT-containing glasses may provide a useful route for developing multifunctional materials in which composition-driven structural changes affect both optical and shielding characteristics.

Another important motivation for investigating these systems is the growing demand for transparent and environmentally safer shielding materials. Conventional shielding media such as lead and concrete are limited by toxicity, high weight, and lack of optical transparency. In contrast, borate glasses containing $\:{\mathrm{B}\mathrm{i}}_{2}{\mathrm{O}}_{3}$ offer high density and high effective atomic number, which favor strong X-ray and gamma-ray attenuation while retaining transparency^8,11^. The introduction of BCZT into such glasses may further enhance photon–matter interactions by introducing highly polarizable cations such as Ba²⁺, Ti⁴⁺, and Zr⁴⁺, while also modifying the local glass structure. This suggests that BCZT/$\:{\mathrm{B}\mathrm{i}}_{2}{\mathrm{O}}_{3}$-containing borate glasses could serve as a promising multifunctional platform for both optical and radiation-related applications.

Recent studies^9,10,12–14^ have demonstrated significant progress in developing multifunctional glass and glass–ceramic systems for optical and radiation shielding applications. However, most of these studies focus primarily on conventional glass hosts such as silicates and phosphates, with limited attention to borate-based systems, despite their superior glass-forming ability, low melting temperatures, and favourable optical properties. In particular, BCZT-containing systems have been extensively investigated for their dielectric, piezoelectric, and structural properties, whereas their integration into borate glass networks for multifunctional optical and radiation-shielding applications remains largely unexplored. Moreover, the existing literature typically reports optical and shielding properties separately, without establishing a clear mechanistic relationship among compositional modification, structural rearrangement, and the simultaneous multifunctional performance.

Therefore, the main research problem addressed in this work is the lack of a systematic understanding of how $\:{\mathrm{B}\mathrm{i}}_{2}{\mathrm{O}}_{3}$ substitution influences the structural evolution of BCZT-containing borate glasses and how these structural changes govern both optical and gamma-ray shielding behavior. The research gap can be summarized in two aspects: (i) the limited exploration of BCZT incorporation within borate glass systems compared to other glass matrices, and (ii) the absence of a comprehensive correlation between $\:{\mathrm{B}\mathrm{i}}_{2}{\mathrm{O}}_{3}$-induced structural modifications and the resulting optical and radiation attenuation properties.

Based on the high atomic number, strong polarizability of Bi³⁺ ions, and the structural role of BCZT-related oxides, we hypothesize that $\:{\mathrm{B}\mathrm{i}}_{2}{\mathrm{O}}_{3}$ incorporation will simultaneously modify the borate network (through BO₄/BO₃ conversion and non-bridging oxygen formation), enhance electronic polarizability, and increase density, leading to improved nonlinear optical response and enhanced gamma-ray shielding efficiency. In addition, this study explores the novel concept of introducing a small fraction of glass former (up to 5 mol%) while preserving the fundamental glass structure and its functional properties, thereby achieving compositional tuning without compromising structural integrity. To the best of our knowledge, this is the first systematic study that establishes a direct structure–property correlation in $\:{\mathrm{B}\mathrm{i}}_{2}{\mathrm{O}}_{3}$-modified borate–BCZT glasses for simultaneous optical and radiation shielding optimization, while demonstrating the feasibility of incorporating a limited amount of glass former without degrading the glass network or its performance. This work goes beyond compositional variation by establishing a clear structure–property–function relationship, demonstrating that $\:{\mathrm{B}\mathrm{i}}_{2}{\mathrm{O}}_{3}$ enables multifunctional optimization within a single glass system.

Accordingly, this study investigates borate–BCZT glass systems with varying. $\:{\mathrm{B}\mathrm{i}}_{2}{\mathrm{O}}_{3}$ content to establish composition–structure–property relationships using density, FTIR, UV–Vis–NIR, and Phy-X analyses. It should be emphasized that the compositional variation is solely based on $\:{\mathrm{B}\mathrm{i}}_{2}{\mathrm{O}}_{3}$ content; therefore, all observed changes are directly correlated with its incorporation, while BCZT acts only as part of the base glass matrix and not as an independent variable. Rather than presenting optical and shielding properties as separate observations, this work aims to demonstrate that $\:{\mathrm{B}\mathrm{i}}_{2}{\mathrm{O}}_{3}$ incorporation acts as a rational design parameter for achieving multifunctional optimization within a single glass system, enabling simultaneous enhancement of photonic, optoelectronic, and radiation shielding performance.

## Experimental work

### Sample preparation

**Fig. 1:**
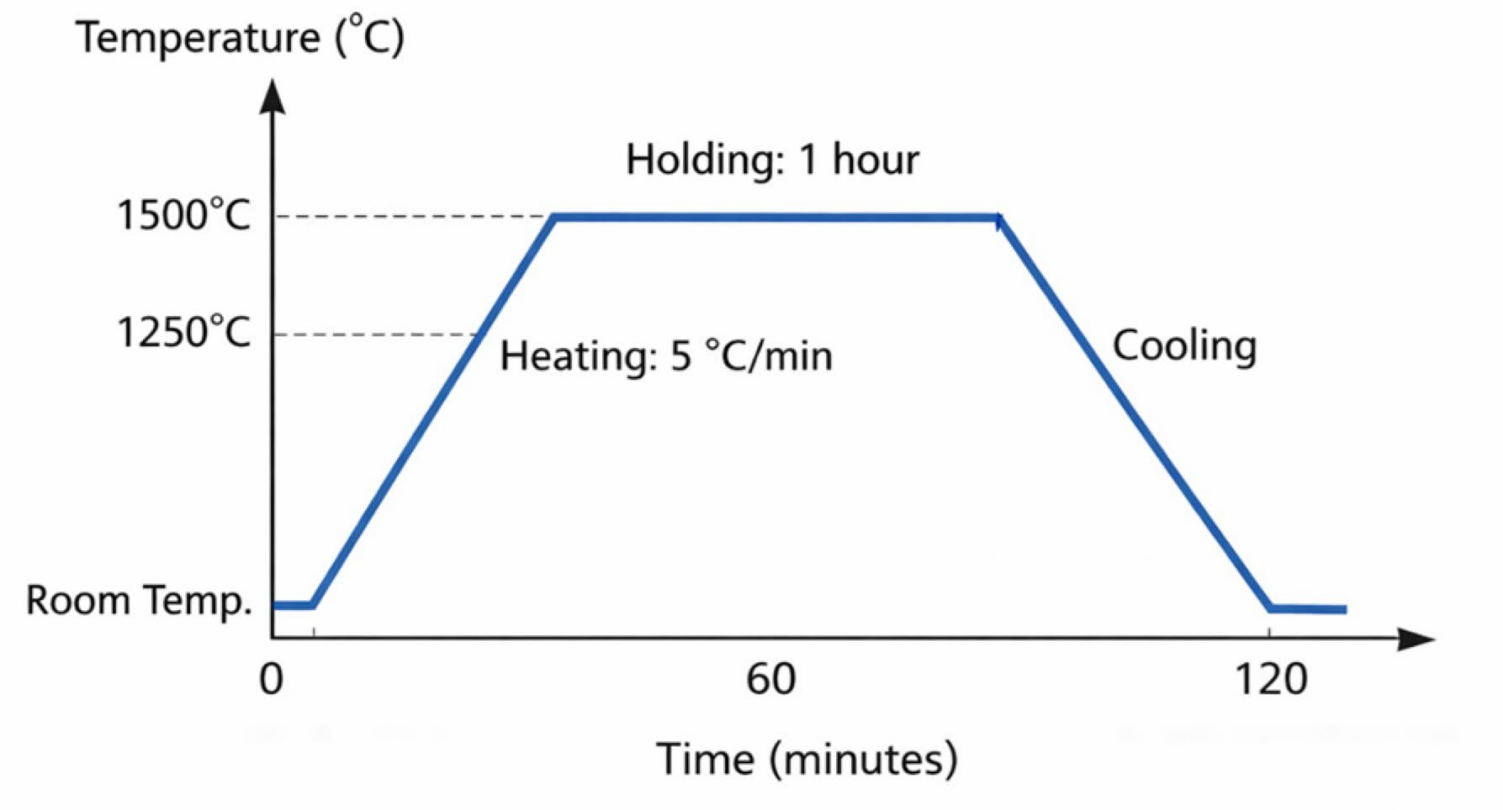
Schematic representation of the thermal profile used for sample preparation.

**Fig. 2:**
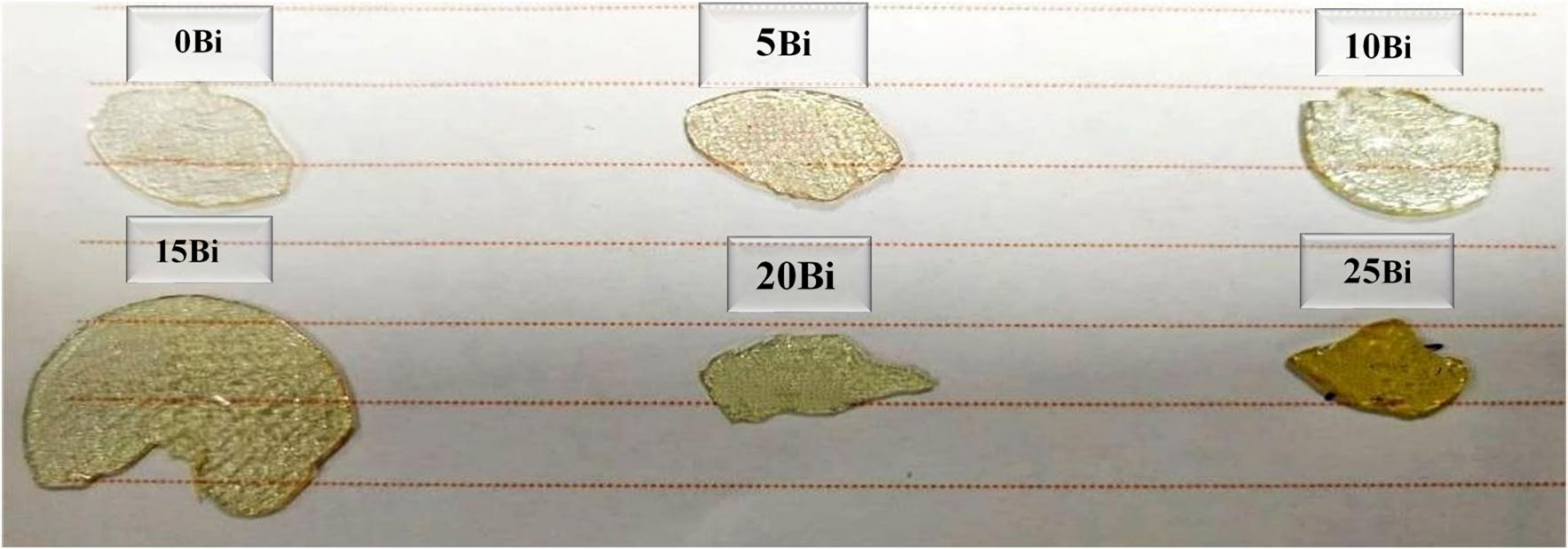
Photograph of the glass samples.

Borate glass samples composed of x$\:{\mathrm{B}\mathrm{i}}_{2}{\mathrm{O}}_{3}$ +$\:\left(30-\mathrm{x}\right){\mathrm{B}}_{2}{\mathrm{O}}_{3}$ + 70 [($\:{\mathrm{B}\mathrm{a}}_{0.85}{\mathrm{C}\mathrm{a}}_{0.15}$)( $\:{\mathrm{T}\mathrm{i}}_{0.9}{\mathrm{Z}\mathrm{r}}_{0.1}$)$\:{\mathrm{O}}_{3}$] in which x = 0–25 mol%, were constructed using the method of melting quenching. High-purity powder was used as the starting material, such as BaC$\:{\mathrm{O}}_{3}$ ($\:\ge\:$99.9 Sigma Aldrich), CaC$\:{\mathrm{O}}_{3}$ ($\:\ge\:$99.9 Alfa Acer), Zr$\:{\mathrm{O}}_{2}$($\:\ge\:$99.9 Sigma Aldrich), Ti$\:{\mathrm{O}}_{2}$ ($\:\ge\:$99.9 Sigma Aldrich), $\:{\mathrm{B}\mathrm{i}}_{2}{\mathrm{O}}_{3}$ (($\:\ge\:$99.9 Sigma Aldrich) and $\:{\mathrm{H}}_{3}\mathrm{B}{\mathrm{O}}_{3}$ ($\:\ge\:$98.5 Nasr Pharmaceutical). The glass samples were prepared using the conventional melt-quenching technique. Appropriate amounts of high-purity raw materials were accurately weighed according to the desired compositions (see Table 1). The powders were thoroughly mixed using an agate mortar to ensure homogeneity. The homogeneous mixture was then transferred to porcelain crucibles and heated under controlled conditions. Figure 1 shows the thermal profile, which consisted of heating the samples from room temperature to the melting range (1250–1500 °C) at a heating rate of approximately 5 °C/min, as shown in Table 2. The melts were maintained at the target temperature for 1 h to ensure complete melting and compositional uniformity, with intermittent stirring to minimize air bubble formation. Finally, the molten samples were rapidly quenched between two stainless steel plates at room temperature to produce amorphous glass. The obtained glasses were transparent, homogenous, and glittering yellow (see Fig. 2).

**Table 1:** Batch composition and calculated weight of raw materials for different glass samples.

Code	$\:{\mathbf{B}\mathbf{i}}_{2}{\mathbf{O}}_{3}$ (mol%)	$\:{\mathbf{B}}_{2}{\mathbf{O}}_{3}$ (mol%)	BCZT (mol%)	$\:{\mathbf{B}\mathbf{i}}_{2}{\mathbf{O}}_{3}$ (g)	$\:{\mathbf{B}}_{2}{\mathbf{O}}_{3}$ (g)	$\:{\mathbf{T}\mathbf{i}\mathbf{O}}_{2}$ (g)	Ba$\:\mathbf{C}{\mathbf{O}}_{3}$ (g)	Ca$\:\mathbf{C}{\mathbf{O}}_{3}$ (g)	$\:{\mathbf{Z}\mathbf{r}\mathbf{O}}_{2}$ (g)	Sum (g)
0 Bi	0	30	70	0	3.092	2.096	4.892	0.438	0.359	10.878
5 Bi	5	25	70	1.942	2.576	2.096	4.892	0.438	0.359	12.304
10 Bi	10	20	70	3.883	2.061	2.096	4.892	0.438	0.359	13.730
15 Bi	15	15	70	5.825	1.546	2.096	4.892	0.438	0.359	15.156
20 Bi	20	10	70	7.766	1.031	2.096	4.892	0.438	0.359	16.586
25 Bi	25	5	70	9.708	0.515	2.096	4.892	0.438	0.359	18.009

**Table 2:** Glass sample codes, composition, and preparation temperature ($\:T)$.

Code	Composition	$\:\boldsymbol{T}$($\:^\circ\:\boldsymbol{C}$)
0 Bi	$\:0{\mathrm{B}\mathrm{i}}_{2}{\mathrm{O}}_{3}$ + 30 $\:{\mathrm{B}}_{2}{\mathrm{O}}_{3}$ + 70[($\:{\mathrm{B}\mathrm{a}}_{0.85}{\mathrm{C}\mathrm{a}}_{0.15}$)( $\:{\mathrm{T}\mathrm{i}}_{0.9}{\mathrm{Z}\mathrm{r}}_{0.1}$)$\:{\mathrm{O}}_{3}$]	1250
5 Bi	$\:5{\mathrm{B}\mathrm{i}}_{2}{\mathrm{O}}_{3}$ + 25 $\:{\mathrm{B}}_{2}{\mathrm{O}}_{3}$ + 70[($\:{\mathrm{B}\mathrm{a}}_{0.85}{\mathrm{C}\mathrm{a}}_{0.15}$)( $\:{\mathrm{T}\mathrm{i}}_{0.9}{\mathrm{Z}\mathrm{r}}_{0.1}$)$\:{\mathrm{O}}_{3}$]	1300
10 Bi	$\:10{\mathrm{B}\mathrm{i}}_{2}{\mathrm{O}}_{3}$ + 20 $\:{\mathrm{B}}_{2}{\mathrm{O}}_{3}$ + 70[($\:{\mathrm{B}\mathrm{a}}_{0.85}{\mathrm{C}\mathrm{a}}_{0.15}$)( $\:{\mathrm{T}\mathrm{i}}_{0.9}{\mathrm{Z}\mathrm{r}}_{0.1}$)$\:{\mathrm{O}}_{3}$]	1350
15 Bi	$\:15{\mathrm{B}\mathrm{i}}_{2}{\mathrm{O}}_{3}$ + 15 $\:{\mathrm{B}}_{2}{\mathrm{O}}_{3}$ + 70[($\:{\mathrm{B}\mathrm{a}}_{0.85}{\mathrm{C}\mathrm{a}}_{0.15}$)( $\:{\mathrm{T}\mathrm{i}}_{0.9}{\mathrm{Z}\mathrm{r}}_{0.1}$)$\:{\mathrm{O}}_{3}$]	1400
20 Bi	$\:20{\mathrm{B}\mathrm{i}}_{2}{\mathrm{O}}_{3}$ + 10 $\:{\mathrm{B}}_{2}{\mathrm{O}}_{3}$ + 70[($\:{\mathrm{B}\mathrm{a}}_{0.85}{\mathrm{C}\mathrm{a}}_{0.15}$)( $\:{\mathrm{T}\mathrm{i}}_{0.9}{\mathrm{Z}\mathrm{r}}_{0.1}$)$\:{\mathrm{O}}_{3}$]	1450
25 Bi	$\:25{\mathrm{B}\mathrm{i}}_{2}{\mathrm{O}}_{3}$ + 5 $\:{\mathrm{B}}_{2}{\mathrm{O}}_{3}$ + 70[($\:{\mathrm{B}\mathrm{a}}_{0.85}{\mathrm{C}\mathrm{a}}_{0.15}$)( $\:{\mathrm{T}\mathrm{i}}_{0.9}{\mathrm{Z}\mathrm{r}}_{0.1}$)$\:{\mathrm{O}}_{3}$]	1500

### Samples characterization

Several experimental methods were utilized in order to distinguish the glass samples. X-ray diffraction (XRD) measurements provided information on glass’s amorphous nature were collected with a $\:{\mathrm{D}}_{8}$ Advance with DAVINCI design (Bruker, Germany), using as X-ray source the Cu Kα radiation (wavelength λ = 1.5418 Å), at 40 kV and 40 mA, a 2θ range of 20–80°, a step size of 0.02°, and a time/step of 0.6 s. A Si zero-background sample holder was used, operated by DIFFRAC, Measurements Center Version V7.3.0 (32Bit) software. The density of the glass samples was determined using Archimedes’ principle, employing toluene as the immersion liquid with a density of 0.867 g/cm³. To ensure measurement accuracy, the sample weights in air and in the immersion liquid were measured five times, and the average values were used to minimize experimental error. The obtained density values were further used to calculate the molar volume (*V*_*m*_) and other related parameters, including Bi ion concentration (N), polaron radius ($\:{r}_{p}$), field strength (F), molar volume of boron atoms ($\:{V}_{m}^{B}$), and the average boron–boron separation (*d*_*B−B*_). FTIR spectra were recorded in the 400–4000 cm⁻¹ wavenumber range at room temperature using a Nicolet 6700 FTIR spectrometer. The samples were prepared by mixing with KBr and pressing into pellets for measurement. The optical absorption spectra were measured at room temperature using a Jenway 6405 UV–Vis–NIR spectrophotometer in the wavelength range of 190–1100 nm with a resolution of 1 nm. Finally, the gamma-ray shielding parameters of the prepared glass samples were evaluated using the Phy-X/PSD software based on their elemental composition and density.

## Results and discussions

### XRD

**Fig. 3:**
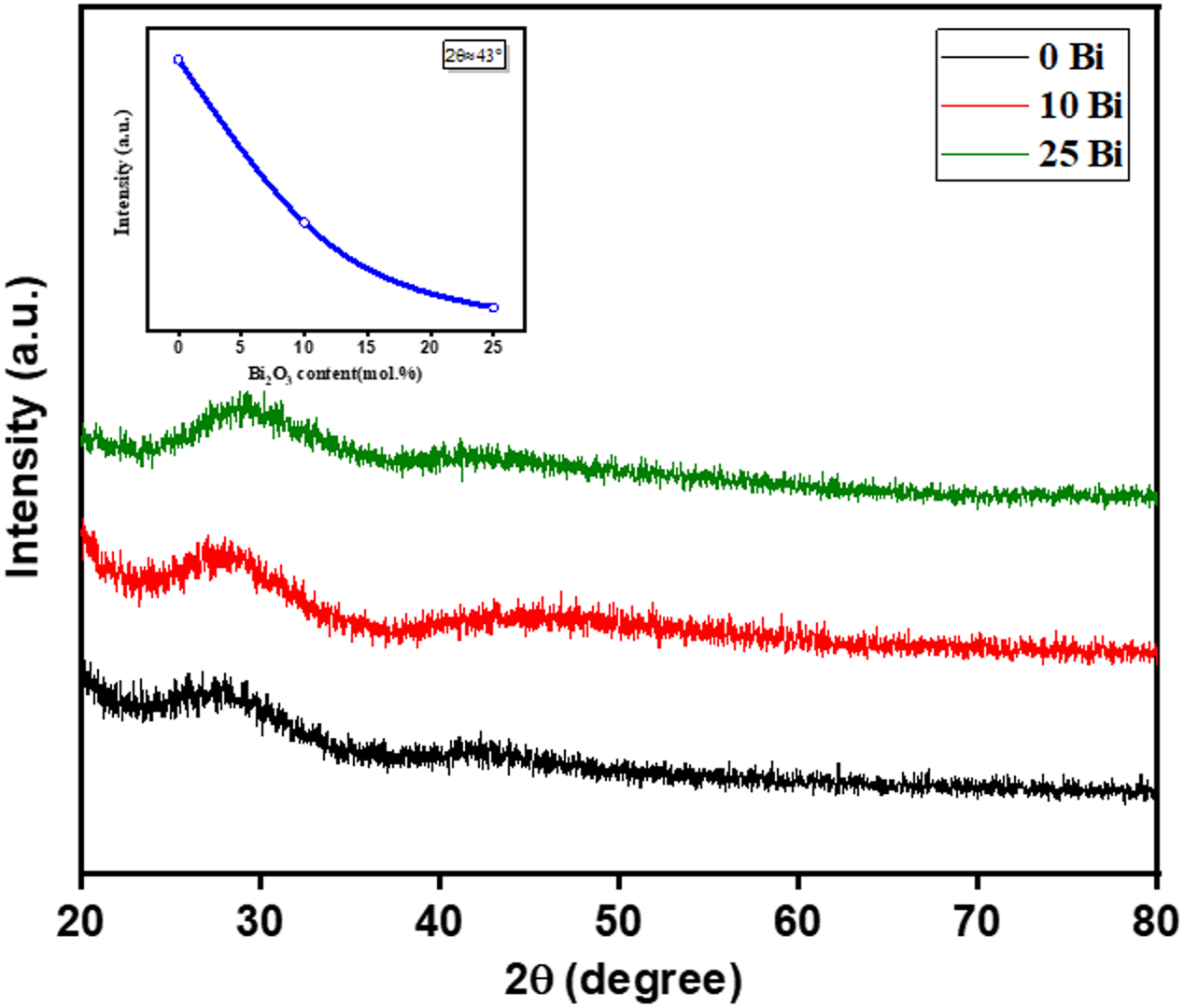
X-ray diffraction of glass samples (0, 10, and 25Bi mol%).

Figure 3 shows the XRD patterns of the synthesized glass samples with varying compositions (x = 0, 10, and 20 mol%). The absence of sharp and well-defined diffraction peaks in all samples confirms the amorphous nature of the prepared glasses. Two broad diffuse humps, centered at approximately 2θ ≈ 27° and 42°, are observed, characteristic of borate glass structures. In addition, a broad feature around 2θ ≈ 45° becomes more pronounced with increasing $\:{\mathrm{B}\mathrm{i}}_{2}{\mathrm{O}}_{3}$ content, indicating enhanced structural disorder and progressive depolymerization of the borate network. The highest intensity is observed for the Bi-free sample (x = 0), while the intensity decreases with increasing $\:{\mathrm{B}\mathrm{i}}_{2}{\mathrm{O}}_{3}$ content. This behavior can be attributed to the gradual substitution of borate-forming units by $\:{\mathrm{B}\mathrm{i}}_{2}{\mathrm{O}}_{3}$, leading to a reduction in the contribution of borate structural units and consequently weakening the diffuse XRD features^15–17^.

### Density ($\:\rho\:$) assessment

Density is a key parameter for investigating structural changes in the glass network. The density (ρ) of the prepared glass samples was determined using Archimedes’ principle according to the following relation:

1 $$\:\rho\:=\frac{{w}_{air}}{{w}_{air}-{w}_{liquid}}{\rho\:}_{liquid}$$

In which $\:{w}_{air}$ and $\:{w}_{liquid}$ represent the weight of the sample in air and in the immersion liquid (toluene), respectively, whereas $\:{\rho\:}_{liquid}$ represents the liquid’s density (0.867 g/$\:{\mathrm{c}\mathrm{m}}^{3}$)^1,18^. Furthermore, the molar volume can be estimated based on this relationship.

2 $$\:{\mathrm{V}}_{\mathrm{m}\:}=\frac{{M}_{w}}{{\uprho\:}}$$

$\:{M}_{w}$ represents the molecular weight of all components. The values of density and molar volume are illustrated in Table 3.

It was observed that the density values increased from 3.622 to 5.788 g/$\:{\mathrm{c}\mathrm{m}}^{3}$ with increasing $\:{\mathrm{B}\mathrm{i}}_{2}{\mathrm{O}}_{3}$ content, with an estimated error of ± 0.011 g/$\:{\mathrm{c}\mathrm{m}}^{3}$. This increase can be attributed to two main factors. First, the substitution of boron oxide, which has a relatively low molecular weight and density (69.62 g/mol and 2.46 g/$\:{\mathrm{c}\mathrm{m}}^{3}$), with bismuth oxide, which possesses a much higher molecular weight and density (465.96 g/mol and 8.9 g/$\:{\mathrm{c}\mathrm{m}}^{3}$), leads to an overall increase in mass per unit volume^15^. In addition, the molar volume of the samples was found to increase from 27.305 to 34.204 $\:{\mathrm{c}\mathrm{m}}^{3}/\mathrm{m}\mathrm{o}\mathrm{l}$ with increasing $\:{\mathrm{B}\mathrm{i}}_{2}{\mathrm{O}}_{3}$ content, with an assessed inaccuracy of ± 0.038 $\:{\mathrm{c}\mathrm{m}}^{3}/\mathrm{m}\mathrm{o}\mathrm{l}$. This behavior is attributed to the incorporation of larger $\:{Bi}^{3+}$ ions (ionic radius ≈ 1.03 Å) in the place of smaller $\:{\mathrm{B}}^{3+}$ ions (ionic radius ≈ 0.27 Å), resulting in the creation of additional free volume within the glass network. Consequently, the glass structure becomes more open, leading to an expansion of the network and a decrease in its compactness^15,19^. Furthermore, FTIR analysis supports this structural modification, where the increase in non-bridging oxygen (NBO) formation indicates the disruption of B–O–B linkages. The generation of NBOs weakens the glass network connectivity and promotes a more open structure. Therefore, the simultaneous increase in density and molar volume reflects a dual effect: mass enhancement due to $\:{\mathrm{B}\mathrm{i}}_{2}{\mathrm{O}}_{3}$ incorporation and structural expansion associated with increased NBO formation and network depolymerization. Several density-related parameters were computed, including $\:{Bi}^{3+}$ ions concentration (N), average distance between the boron atoms (<$\:{d}_{B-B}>)$, Polaron radius of Bi ($\:{r}_{p}$) and the field strength of Bi (F). The following formula was used to estimate these parameters^15,20^.

3 $$\:{N}_{Bi}=mol\%\:of\:Bi\:\frac{{\rho\:N}_{A}}{{M}_{w}}$$

4 $$\:{r}_{p}=\left(\frac{1}{2}\right)({\frac{\pi\:}{6N})}^{1/3}$$

5 $$\:F=\frac{Z}{{{r}_{p}}^{2}}$$

6 $$\:{<d}_{B-B}>\:={\left(\frac{{V}_{m}^{b}}{{N}_{A}}\:\right)}^{1/3}$$

7 $$\:\:\:{V}_{m}^{b}=\frac{{V}_{m\:}}{2\left(1-{X}_{B}\right)}$$

Where $\:{\mathrm{N}}_{\mathrm{A}}$, $\:{V}_{m}^{b}$, $\:{X}_{B}$, and Z represent Avogadro’s number, boron atoms’ molar volume, mole fraction of $\:{\mathrm{B}\mathrm{i}}_{2}{\mathrm{O}}_{3}$, and the atomic number of Bi atoms, respectively. The values of these parameters were recorded, see Table 3. According to the obtained results, the concentration of Bi³⁺ ions ($\:{N}_{Bi}$) increases significantly with increasing $\:{\mathrm{B}\mathrm{i}}_{2}{\mathrm{O}}_{3}$ content, ranging from 1.023 $\:\times\:{10}^{21}$ up to 4.401 $\:\times\:{10}^{21}$ ions/$\:{\mathrm{c}\mathrm{m}}^{3}$. This trend is expected due to the greater incorporation of bismuth oxide, which contributes to an overall increase in the glass density^18^. In contrast, the polaron radius (rₚ) decreases from 0.399 to 0.245 nm with increasing $\:{\mathrm{B}\mathrm{i}}_{2}{\mathrm{O}}_{3}$ content. This reduction can be attributed to enhanced electron localization resulting from the formation of trapping centers and stronger electron–phonon interactions. Consequently, small polarons with limited spatial extent are formed, leading to a decrease in $\:{r}_{p}$^21,22^. Furthermore, an increase in Bi³⁺ ion concentration enhances the field strength (F). This behavior is associated with a structural rearrangement within the borate network, particularly the transformation of BO₃ units into BO₄ units, as confirmed by FTIR analysis. This transformation strengthens the bonding environment and increases the electrostatic field around Bi³⁺ ions^18,22^. Additionally, the incorporation of $\:{\mathrm{B}\mathrm{i}}_{2}{\mathrm{O}}_{3}$ induces local structural rearrangement, which leads to a decrease in the average boron–boron separation (<$\:{d}_{B-B}>$(. This local compaction occurs despite the overall expansion of the glass network, as reflected by the increase in molar volume^15,18^. Therefore, the incorporation of $\:{\mathrm{B}\mathrm{i}}_{2}{\mathrm{O}}_{3}$ results in a dual structural effect: global network expansion accompanied by local structural rearrangement and reduced interatomic distances in specific regions.

**Table 3:** Density (), molar volume (*V*_*m*_), concentration of Bi ions ($\:{N}_{Bi}$), Polaron raduis of Bi ($\:{r}_{p}$), field strength of Bi (F), boron atom molar volume( $\:{V}_{m}^{B}$), and average boron-boron separation (*d*_*B−B*_).

Sample (mol%)	$\:\rho\:$ (g/cm^3^) ± 0.01	$\:{\boldsymbol{V}}_{\boldsymbol{m}}$ (cm^3^/mol) ± 0.03	$\:{\boldsymbol{N}}_{\boldsymbol{B}\boldsymbol{i}}$ (×10^21^/cm^3^) ± 0.002 × 10^21^	$\:{\boldsymbol{r}}_{\boldsymbol{p}}$ (nm) ± 0.0002	F (cm^− 2^ × 10^17^) ± 1.9 × 10^14^	$\:{\boldsymbol{V}}_{\boldsymbol{m}}^{\boldsymbol{B}}$ (cm^3^/mol) ± 0.04	d_B−B_ (nm) ± 0.0001
0 Bi	3.62	27.3	-	-	-	19.5	0.319
5 Bi	4.07	29.1	1.03	0.39	0.51	19.4	0.318
10 Bi	4.52	30.6	1.96	0.32	0.80	19.1	0.316
15 Bi	5.03	31.4	2.87	0.28	1	18.5	0.313
20 Bi	5.26	33.8	3.55	0.26	1.19	18.80	0.314
25 Bi	5.78	34.2	4.40	0.24	1.36	18	0.310

### FT-IR spectra

**Fig. 4:**
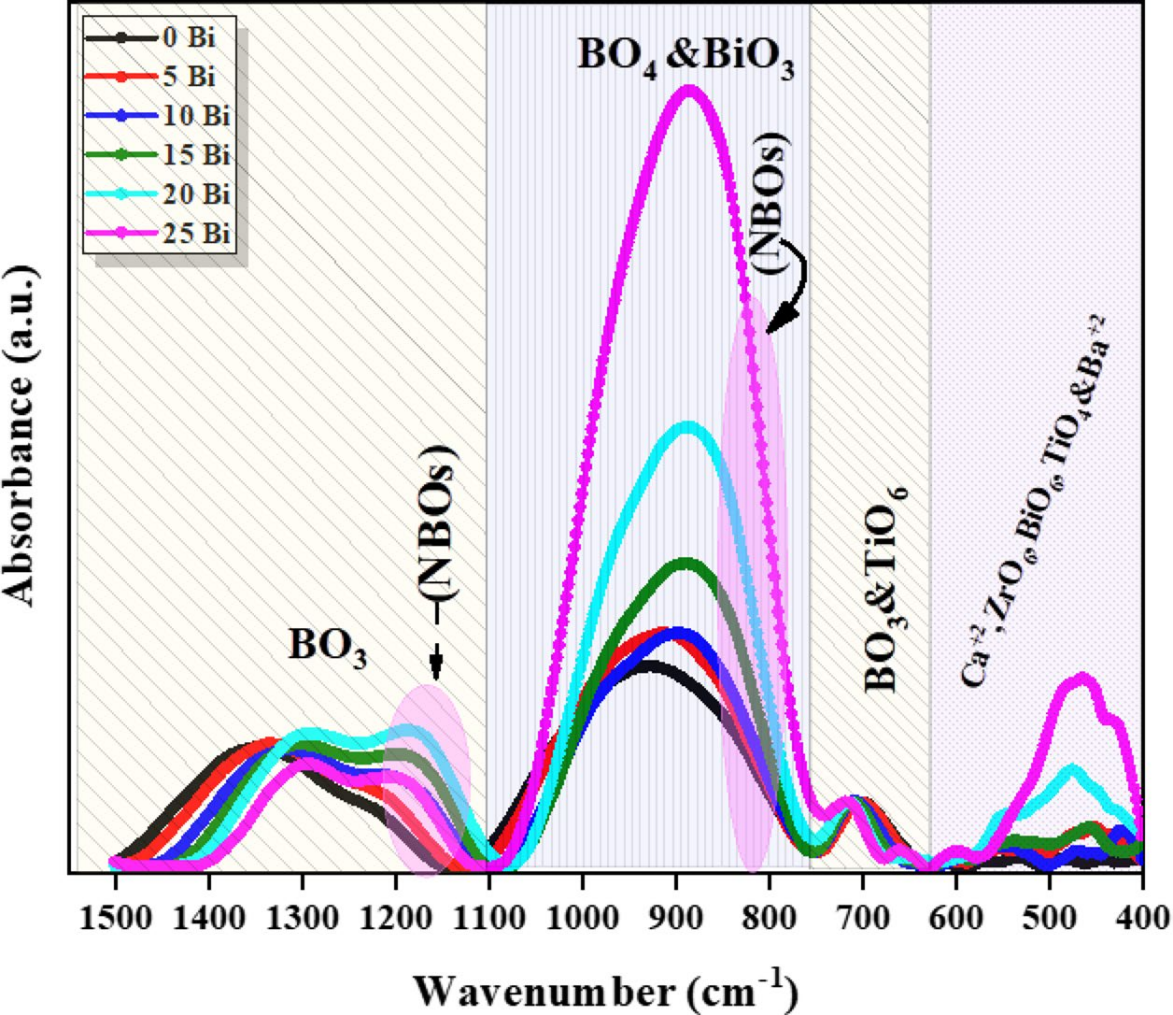
FTIR spectra for all glass samples.

**Fig. 5:**
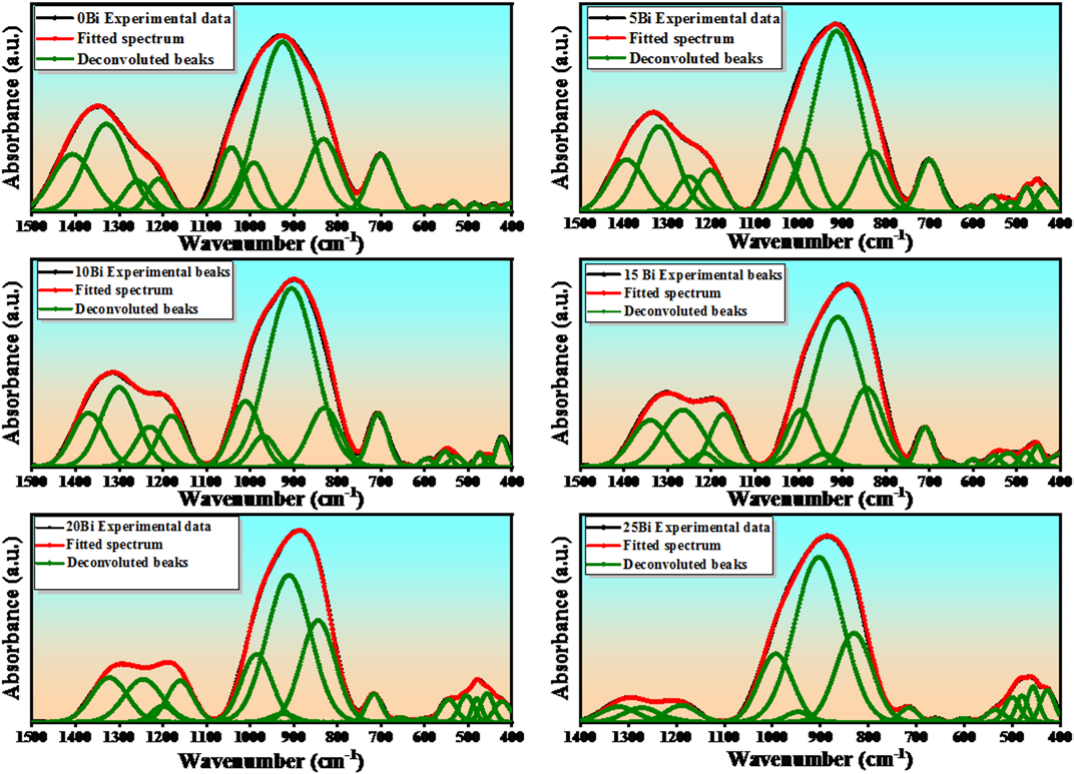
Deconvolution of the prepared glass samples.

The structural investigation of the glass samples was carried out using FTIR spectroscopy, an effective technique for identifying structural units and functional groups within the glass network. Figure 4 presents the FTIR spectra of the prepared glasses in the wavenumber range of 400–1500 $\:{\mathrm{c}\mathrm{m}}^{-1}$. The spectra can be divided into four main regions. The first region (400–600 cm⁻¹) is attributed to the vibration modes of metal cations such as Ca²⁺, Ba²⁺, Ti⁴⁺, and Zr⁴⁺. The second region (600–760 cm⁻¹) corresponds to bending vibrations of $\:{\mathrm{B}\mathrm{O}}_{3}$ units. The third region (760–1000 cm⁻¹) is associated with the stretching vibrations of $\:{\mathrm{B}\mathrm{O}}_{4}$ units, while the fourth region (1060–1500 cm⁻¹) is assigned to the stretching vibrations of $\:{\mathrm{B}\mathrm{O}}_{3}$ units. It is important to note that the FTIR spectra exhibit broad, overlapping bands characteristic of amorphous glass. Therefore, Gaussian deconvolution was applied to resolve these overlapping bands and accurately identify the individual vibrational contributions. Figure 5 shows the deconvoluted spectra, and the peak positions along with their corresponding assignments are summarized in Table 4. The region located in range (400–600 $\:{\mathrm{c}\mathrm{m}}^{-1}$) was attributed to vibrations of metallic cations such as $\:{\mathrm{C}\mathrm{a}}^{2+}$^23,24^, vibration of octahedron $\:{{[\mathrm{Z}\mathrm{r}\mathrm{O}}_{6}\:]}^{2-}$^25,26^, Bi-O stretching vibration in the octahedral $\:{\mathrm{B}\mathrm{i}\mathrm{O}}_{6}$^18,26^ and vibration of $\:{\mathrm{B}\mathrm{a}}^{2+}$ and $\:{\mathrm{T}\mathrm{i}\mathrm{O}}_{4}$ structural units^3,25,27^. The region located at 637$\:{\mathrm{c}\mathrm{m}}^{-1}$ was attributed to B-O-B bending vibration in borate ring angle distortions and vibration of Ti-O-Ti bridge associated with $\:{\mathrm{T}\mathrm{i}\mathrm{O}}_{6}$^3,25,28^. The region 705 $\:{\mathrm{c}\mathrm{m}}^{-1}$ matching the bending vibrations of the B-O-B in asymmetric bridge-oxygen trigonal $\:{\mathrm{B}\mathrm{O}}_{3}$ units^2,28^. The bands referred to around 834 $\:{\mathrm{c}\mathrm{m}}^{-1}$ ascribed to the stretching vibration of Bi-O-Bi in $\:{\mathrm{B}\mathrm{i}\mathrm{O}}_{3}$ units and/or the B–O symmetric stretching vibrations of $\:{\mathrm{B}\mathrm{O}}_{4}$ units, but the bands at the range (903–1043 $\:{\mathrm{c}\mathrm{m}}^{-1}$) could be allocated to B–O bond stretching of tetrahedral $\:{\mathrm{B}\mathrm{O}}_{4}$ units^2,3,29^. Eventually, the band at 1185 $\:{\mathrm{c}\mathrm{m}}^{-1}$ could occur because of the symmetric stretching vibrations of ($\:{\mathrm{N}\mathrm{B}\mathrm{O})}_{{\mathrm{B}\mathrm{O}}_{3}}$in trigonal $\:{\mathrm{B}\mathrm{O}}_{3}$, while the regions (1200–1400 $\:{\mathrm{c}\mathrm{m}}^{-1}$) associated with asymmetric stretching vibrations of B-O bonds in the $\:{\mathrm{B}\mathrm{O}}_{3}$ units^2,3,28^. As shown in Fig. 5, with increasing $\:{\mathrm{B}\mathrm{i}}_{2}{\mathrm{O}}_{3}$ content, the intensity of the bands in the regions 600–760 cm⁻¹ and 1060–1500 cm⁻¹ decreases, while the intensity of the band in the range 760–1000 cm⁻¹ increases. This behavior indicates a progressive structural transformation from $\:{\mathrm{B}\mathrm{O}}_{3}$ to $\:{\mathrm{B}\mathrm{O}}_{4}$ units within the glass network. The decrease in the intensity of $\:{\mathrm{B}\mathrm{O}}_{3}$-related bands, accompanied by the enhancement of BO₄ bands, suggests that the incorporation of $\:{\mathrm{B}\mathrm{i}}_{2}{\mathrm{O}}_{3}$, acting as a network modifier, promotes the conversion of trigonal borate units into tetrahedral ones. This transformation is associated with the formation of non-bridging oxygens (NBOs), resulting from the breaking of B–O–B linkages^18^. Furthermore, the observed shift of BO₃-related bands toward lower wavenumbers with increasing $\:{\mathrm{B}\mathrm{i}}_{2}{\mathrm{O}}_{3}$ content indicates the transformation of metaborate and orthoborate units into more polymerized $\:{\mathrm{B}\mathrm{O}}_{4}$-containing structural groups. Consequently, the concentration of NBOs increases with increasing $\:{\mathrm{B}\mathrm{i}}_{2}{\mathrm{O}}_{3}$ content^29^. To gain deeper insight into the compositional changes of the glass network, the fractions of $\:{\mathrm{B}\mathrm{O}}_{4}$ units ($\:{N}_{4}$), $\:{\mathrm{B}\mathrm{O}}_{3}$ units ($\:{\mathrm{N}}_{3}$), and NBOs were estimated from the deconvoluted FTIR spectra by calculating the area under the corresponding peaks. These parameters were evaluated using the following relations^1,5^:

8 $$\:{N}_{4}=\frac{{A}_{{BO}_{4}}}{{A}_{{BO}_{4}}+{A}_{{BO}_{3}}}$$

9 $$\:{N}_{3}=\:\frac{{A}_{{BO}_{3}}}{{A}_{{BO}_{4}}+{A}_{{BO}_{3}}}$$

10 $$\:{\mathrm{N}\mathrm{B}\mathrm{O}}_{\mathrm{s}}=\frac{{\mathrm{N}\mathrm{B}\mathrm{O}}_{{\mathrm{B}\mathrm{O}}_{4}}+{\mathrm{N}\mathrm{B}\mathrm{O}}_{{\mathrm{B}\mathrm{O}}_{3}}}{{A}_{{BO}_{4}}+{A}_{{BO}_{3}}}$$

**Fig. 6:**
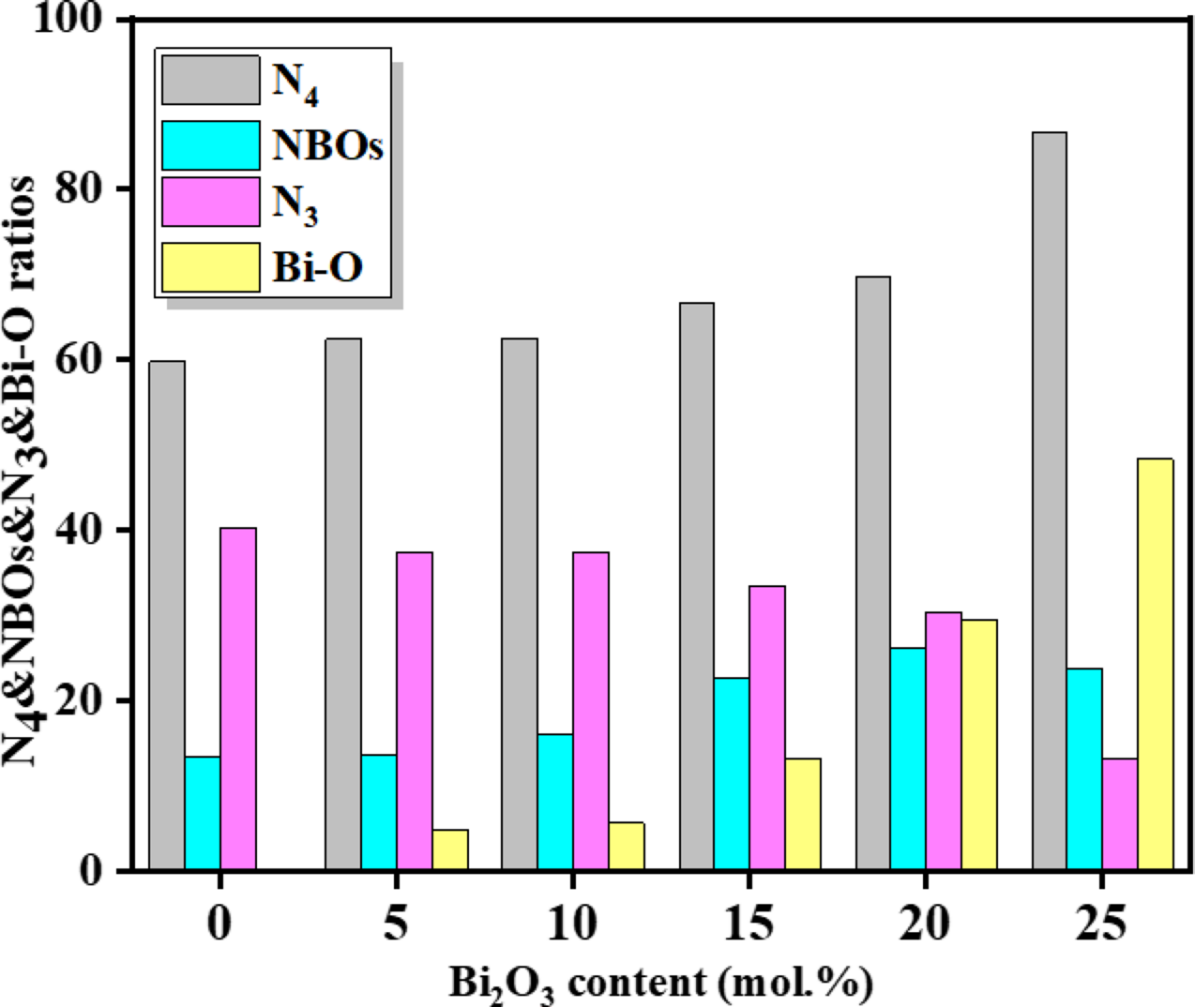
Various values of $\:{N}_{4}$, $\:{N}_{3}$, $\:{NBO}_{s}$ and Bi ratios represented as a function of $\:{Bi}_{2}{O}_{3}$ content across the glasses.

The parameters $\:{A}_{{\mathrm{BO}}_{4}}$ and $\:{A}_{{\mathrm{BO}}_{3}}$ represent the integrated areas of the peaks corresponding to $\:{\mathrm{BO}}_{4}$ and $\:{\mathrm{BO}}_{3}$ structural units, respectively. Meanwhile, $\:{\mathrm{NBO}}_{\left({\mathrm{BO}}_{4}\right)}$ and $\:{\mathrm{NBO}}_{\left({\mathrm{BO}}_{3}\right)}$ denote the areas associated with non-bridging oxygen (NBO) contributions, identified at approximately 844 cm⁻¹ in the BO₄ region and 1160 cm⁻¹ in the $\:{\mathrm{BO}}_{3}$ region, respectively. Based on the calculated values, the fraction of BO₄ units ($\:{\mathrm{N}}_{4}$) increased significantly from 60% to 87%, while the fraction of $\:{\mathrm{BO}}_{3}$ units ($\:{\mathrm{N}}_{3}$) decreased from 40% to 13%. In parallel, the NBO fraction (NBOₛ) increased from 13% to 26%, as illustrated in Fig. 6. The observed increase in the BO₄ fraction ($\:{\mathrm{N}}_{4}$) and the corresponding decrease in the $\:{\mathrm{B}\mathrm{O}}_{3}$ fraction ($\:{\mathrm{N}}_{3}$) with increasing $\:{\mathrm{B}\mathrm{i}}_{2}{\mathrm{O}}_{3}$ content, it clearly reflects the modifying role of $\:{\mathrm{B}\mathrm{i}}_{2}{\mathrm{O}}_{3}$ within the borate network. The incorporation of $\:{\mathrm{B}\mathrm{i}}_{2}{\mathrm{O}}_{3}$ introduces additional oxygen ions, which promote the conversion of trigonal $\:{\mathrm{B}\mathrm{O}}_{3}$ units into a tetrahedral $\:{\mathrm{B}\mathrm{O}}_{4}$ units, indicating a localized increase in network connectivity (structural polymerization). Simultaneously, the enhancement of NBO-related bands confirms the partial disruption of B–O–B linkages and the generation of non-bridging oxygens. Importantly, these processes are not contradictory but occur concurrently as a result of $\:{\mathrm{B}\mathrm{i}}_{2}{\mathrm{O}}_{3}$-induced structural reorganization, where the redistribution of oxygen and borate units leads to the coexistence of increased $\:{\mathrm{B}\mathrm{O}}_{4}$ connectivity and NBO formation within different local environments of the glass network. In addition, Bi³⁺ ions participate in the glass network by forming structural units such as $\:{\mathrm{B}\mathrm{i}\mathrm{O}}_{3}$ and $\:{\mathrm{B}\mathrm{i}\mathrm{O}}_{6}$, suggesting that bismuth can act as both a network modifier and, to some extent, an intermediate former. Furthermore, the FTIR spectra exhibit a distinct absorption band around 514 cm⁻¹, assigned to Bi–O stretching vibrations. The integrated area of this band increases systematically (from 0% to ~ 48%) with increasing $\:{\mathrm{B}\mathrm{i}}_{2}{\mathrm{O}}_{3}$ content (seen Fig. 6), confirming the progressive incorporation of bismuth into the glass network and the formation of Bi–O structural units^5,29^. These structural modifications lead to the depolymerization of the borate network and the formation of a more open, disordered structure. Such changes significantly influence the physical and functional properties of the glasses, including their optical, dielectric, and thermal behavior.

**Table 4:** IR absorption band peak assignments and positions for the glass samples.

Peak No.			Wavenumber $\:{\mathbf{c}\mathbf{m}}^{-1}$				Assignment (vibration modes of the bonds in the structural units)
	0Bi	5Bi	10Bi	15Bi	20Bi	25Bi	
**1**	400	435	423	400	422	427	Vibrations of metallic cations $\:\left[{\mathrm{C}\mathrm{a}}^{2+}\right]$ ^23,24^
**2**	443	454	454	453	457	457	Vibrations of octahedron $\:{{[\mathrm{Z}\mathrm{r}\mathrm{O}}_{6}\:]}^{2-}$^25,26^
**3**	485	477	474	579	480	480	stretching vibration of Bi–O in the octahedral $\:{\mathrm{B}\mathrm{i}\mathrm{O}}_{6}$ ^18,26^
**4**	517	517	528	520	504	500	Symmetrical stretching vibration of Bi–O–Bi bond^3,25,27^
**5**	535	533	553	552	545	535	vibration the metal cations of $\:{\mathrm{B}\mathrm{a}}^{2+}$ and $\:{\mathrm{T}\mathrm{i}\mathrm{O}}_{4}$ units^3,25,28^
**6**	570	558	595	600	600	600	Symmetrical stretching vibration of the Bi–O–Bi bond^30^
**7**	605	605	640	659	655	660	vibration of Ti–O–Ti bridge connected with $\:{\mathrm{T}\mathrm{i}\mathrm{O}}_{6}$ /B–O–B bending vibration in borate ring distortions^3,25,28^
**8**	700	700	706	710	716	716	asymmetric bond bending vibrations of B–O–B of bridge-oxygen in trigonal $\:{\mathrm{B}\mathrm{O}}_{3}$ units^2,3^
**9**	832	845	838	844	844	830	NBOs in $\:{\mathrm{B}\mathrm{O}}_{4}$ units /stretching vibration of Bi–O–Bi bond in $\:{\mathrm{B}\mathrm{i}\mathrm{O}}_{3}$ units^2,29^
**10**	925	929	914	910	911	903	B–O bond stretching of tetrahedral $\:{\mathrm{B}\mathrm{O}}_{4}$ units^2,3,28,18^
**11**	993	982	973	945	927	949	B–O bond stretching of tetrahedral $\:{\mathrm{B}\mathrm{O}}_{4}$ units^2,3,28,1^
**12**	1043	1020	1010	995	985	993	B–O bond stretching of tetrahedral $\:{\mathrm{B}\mathrm{O}}_{4}$ units^2,3,28,1^
**13**	1210	1203	1180	1172	1160	1189	B–O bond asymmetric stretching relaxation of (NBOs) in trigonal $\:{\mathrm{B}\mathrm{O}}_{3}$ units^2,3,28^
**14**	1329	1251	1230	1217	1200	1222	B–O bond asymmetric stretching relaxation in trigonal $\:{\mathrm{B}\mathrm{O}}_{3}$ units^2,3,28^
**15**	1258	1320	1300	1265	1245	1272	B–O bond asymmetric stretching relaxation in trigonal $\:{\mathrm{B}\mathrm{O}}_{3}$ units^2,3,28^
**16**	1407	1394	1370	1340	1322	1320	B–O bond asymmetric stretching relaxation in trigonal $\:{\mathrm{B}\mathrm{O}}_{3}$ units^2,3,28^

### UV-visible (UV-Vis) spectroscopy

**Fig. 7:**
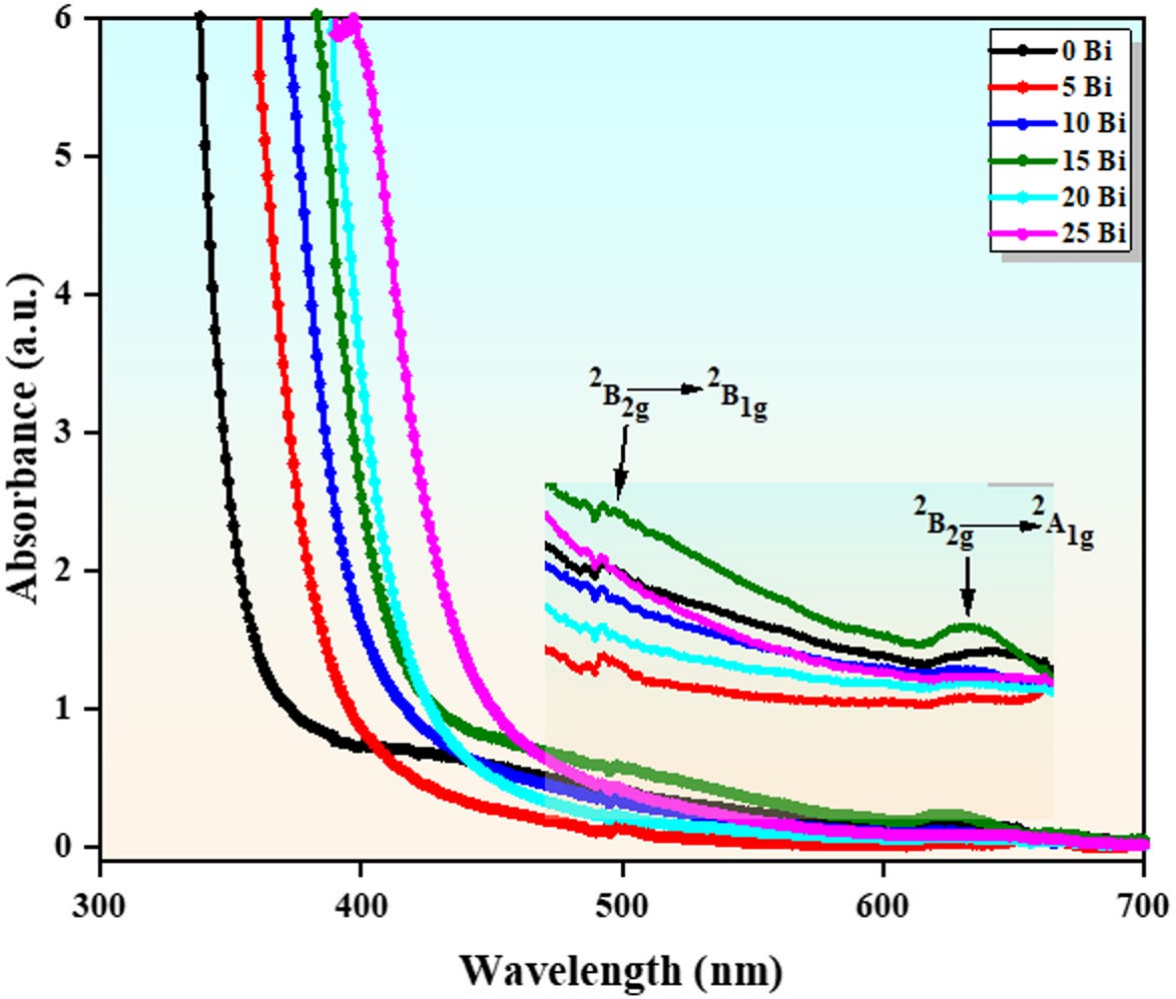
Optical absorption spectra represented in the form of a function of $\:{Bi}_{2}{O}_{3}$ content across the glasses.

UV–Vis spectroscopy is a powerful technique for analyzing the optical properties of glass materials, including the optical absorption edge and band gap energy. Figure 7 presents the UV–visible absorption spectra of the investigated glass samples in the 200–700 nm wavelength range. As shown in the figure, no sharp absorption edge is observed, indicating the amorphous nature of the samples, which is consistent with the XRD results^24,4^. The absorption edge shifts from approximately 360 nm to 445 nm with increasing $\:{\mathrm{B}\mathrm{i}}_{2}{\mathrm{O}}_{3}$ content, reflecting a red shift toward longer wavelengths. This shift indicates a reduction in the optical band gap energy, attributed to structural and electronic modifications induced by $\:{\mathrm{B}\mathrm{i}}_{2}{\mathrm{O}}_{3}$ incorporation.

In addition, two distinct absorption bands are observed at approximately 498 nm and 623 nm, which are assigned to the d–d transitions of $\:{\mathrm{T}\mathrm{i}}^{3+}$ ions, corresponding to the transitions, $\:{}^{2}{\mathrm{B}}_{2\mathrm{g}}\to\:{}^{2}{\mathrm{B}}_{1\mathrm{g}}$ and $\:{}^{2}{\mathrm{B}}_{2\mathrm{g}}\to\:$ $\:{}^{2}{\mathrm{A}}_{1\mathrm{g}}$ respectively^31,32^. These transitions confirm the presence of Ti³⁺ ions with a 3d¹ electronic configuration. In an octahedral coordination environment, the degeneracy of the 3d orbitals is lifted due to crystal field splitting into lower-energy $\:{t}_{2g}$and higher-energy $\:{e}_{g}$ levels. The primary optical transition of $\:{\mathrm{T}\mathrm{i}}^{3+}$involves the excitation of the single 3d electron from the $\:{t}_{2g}$ to the $\:{e}_{g}$ level, resulting in a broad absorption band in the visible region^33^. The position and intensity of these bands are influenced by the glass composition, ligand field strength, and local structural environment.

#### Ligand field

According to Tanabe–Sugano theory, Ti³⁺ ions possess a 3d¹ electronic configuration with a ground state term symbol of $\:{}^{2}\mathrm{D}$, which is fivefold degenerate. In an octahedral crystal field, this degeneracy splits into two levels: a lower-energy $\:{}^{2}{\mathrm{T}}_{2\mathrm{g}}$ state and a higher-energy $\:{}^{2}{\mathrm{E}}_{\mathrm{g}}$ state. Consequently, a single electronic transition $\:{}^{2}{\mathrm{T}}_{2\mathrm{g}}\to\:$ $\:{}^{2}{\mathrm{E}}_{\mathrm{g}}$ is expected, corresponding to the crystal field splitting energy (10Dq)^32^. However, due to the Jahn–Teller effect, the degeneracy of the $\:{}^{2}{\mathrm{T}}_{2\mathrm{g}}$ ground state is partially lifted, leading to a distortion from perfect octahedral symmetry. As a result, the $\:{}^{2}{\mathrm{T}}_{2\mathrm{g}}$level splits into $\:{}^{2}{\mathrm{B}}_{2\mathrm{g}}$and $\:{}^{2}{\mathrm{E}}_{\mathrm{g}}$states, while the excited $\:{}^{2}{\mathrm{E}}_{\mathrm{g}}$ level further splits into $\:{}^{2}{\mathrm{B}}_{1\mathrm{g}}$ and $\:{}^{2}{\mathrm{A}}_{1\mathrm{g}}$states under tetragonal symmetry. This distortion gives rise to multiple electronic transitions instead of a single transition. Accordingly, three possible absorption bands can be observed, corresponding to the transitions $\:{}^{2}{\mathrm{B}}_{2\mathrm{g}}\to\:{}^{2}{\mathrm{E}}_{\mathrm{g}}$, $\:{}^{2}{\mathrm{B}}_{2\mathrm{g}}\to\:{}^{2}{\mathrm{B}}_{1\mathrm{g}}$ and $\:{}^{2}{\mathrm{B}}_{2\mathrm{g}}\to\:$ $\:{}^{2}{\mathrm{A}}_{1\mathrm{g}}$ categorized under $\:{v}_{3}$, $\:{v}_{2}$ and $\:{v}_{1}$, respectively^24^. Due to the relatively weak spin–orbit coupling in the 3d¹ configuration, additional splitting of energy levels may occur, leading to closely spaced absorption bands. In practice, two prominent absorption bands are often observed for distorted Ti³⁺ ions, in addition to the fundamental transition associated with the octahedral field^32^. The crystal field splitting parameter (10Dq) for distorted Ti³⁺ ions can be estimated from the position of the $\:{}^{2}{\mathrm{B}}_{2\mathrm{g}}\to\:{}^{2}{\mathrm{B}}_{1\mathrm{g}}$transition band, as described by the following relation^34^:

11 $$\:{v}_{2}:{}^{2}{\mathrm{B}}_{2\mathrm{g}}\to\:{}^{2}{\mathrm{B}}_{1\mathrm{g}}=\:10\mathrm{D}\mathrm{q}$$

**Fig. 8:**
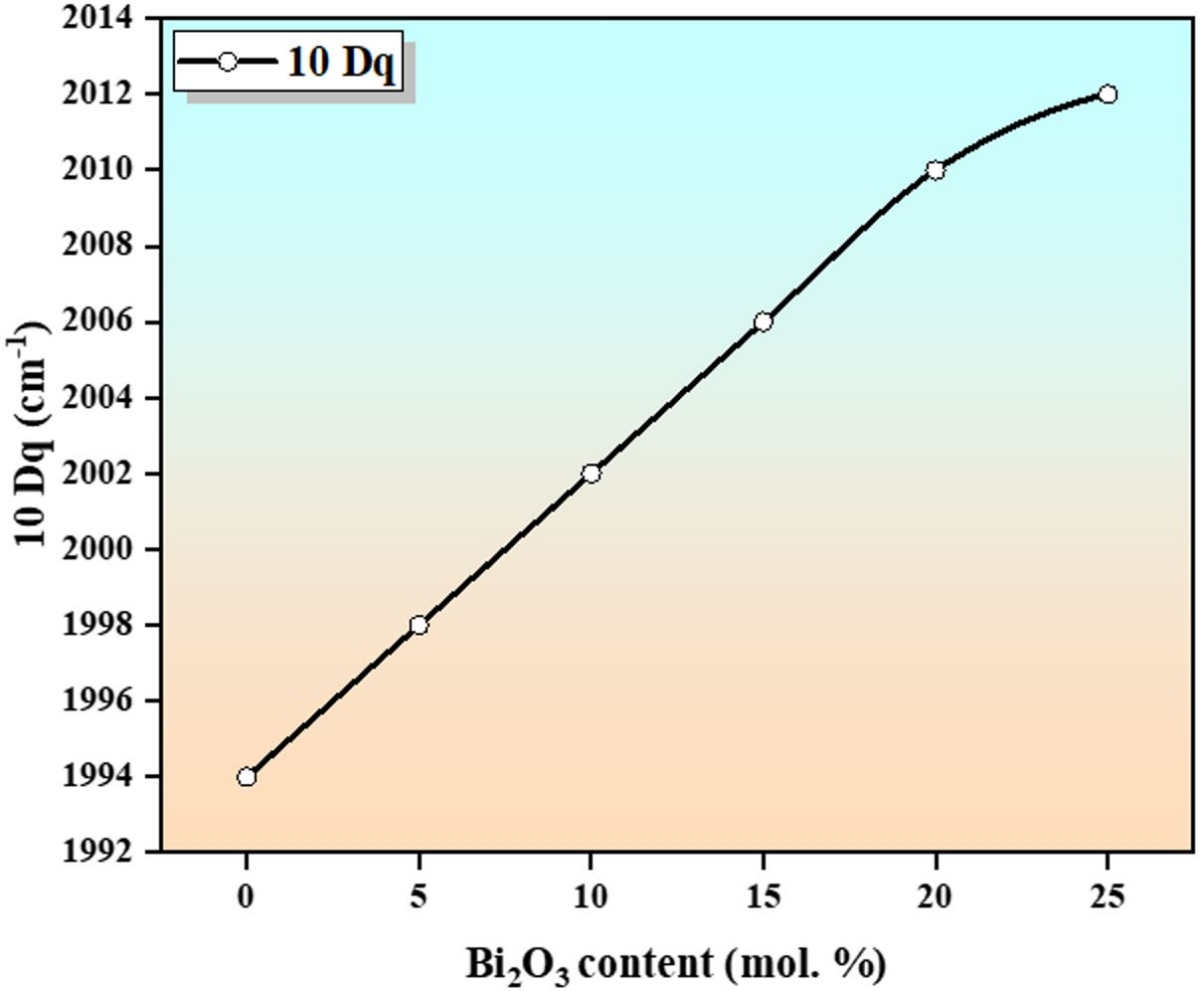
Ligand field strength represented as one of the functions of $\:{Bi}_{2}{O}_{3}$ content.

The values of 10Dq are listed in Table 5, and the results show a systematic increase with increasing $\:{\mathrm{B}\mathrm{i}}_{2}{\mathrm{O}}_{3}$ content, as illustrated in Fig. 8. This behavior can be explained by several interrelated structural and electronic factors. The incorporation of $\:{\mathrm{B}\mathrm{i}}^{3+}$ions, characterized by their large ionic radii and high polarizability, modify the local glass structure and enhance the matrix’s overall polarizability. This, in turn, strengthens the electrostatic interaction between oxygen ligands and $\:{\mathrm{T}\mathrm{i}}^{3+}$ ions, leading to an increase in crystal field splitting^1^. Moreover, the substitution of $\:{\mathrm{B}}_{2}{\mathrm{O}}_{3}$ by $\:{\mathrm{B}\mathrm{i}}_{2}{\mathrm{O}}_{3}$ promotes the formation of non-bridging oxygens (NBOs), which interact more strongly with $\:{\mathrm{T}\mathrm{i}}^{3+}$ ions. This results in improved coordination and the formation of a more regular $\:{\mathbf{T}\mathbf{i}\mathbf{O}}_{6}$ octahedral environment, thereby enhancing the symmetry and strength of the ligand field. In addition, the incorporation of $\:{\mathrm{B}\mathrm{i}}_{2}{\mathrm{O}}_{3}$ induces local structural rearrangement within the glass network. This rearrangement may lead to a slight shortening of Ti–O bond lengths, further strengthening the electrostatic interaction between $\:{\mathrm{T}\mathrm{i}}^{3+}$ ions and surrounding ligands, and consequently increasing 10Dq^35^. Furthermore, the presence of heavy $\:{\mathrm{B}\mathrm{i}}^{3+}$ ions promote local ordering and reduce distortion in $\:{\mathbf{T}\mathbf{i}\mathbf{O}}_{6}$ units, resulting in a more stable and symmetric octahedral field with reduced ligand field asymmetry. The presence of $\:{\mathbf{B}\mathbf{a}}^{2+}$ and $\:{\mathbf{C}\mathbf{a}}^{2+}$ ions also influence the Ti–O bond environment through electrostatic interactions, leading to more symmetric coordination and an enhanced ligand field strength^35^. Overall, these combined structural and electronic effects lead to a systematic increase in 10Dq with increasing $\:{\mathrm{B}\mathrm{i}}_{2}{\mathrm{O}}_{3}$ content.

#### Optical band gap and band tail

**Fig. 9:**
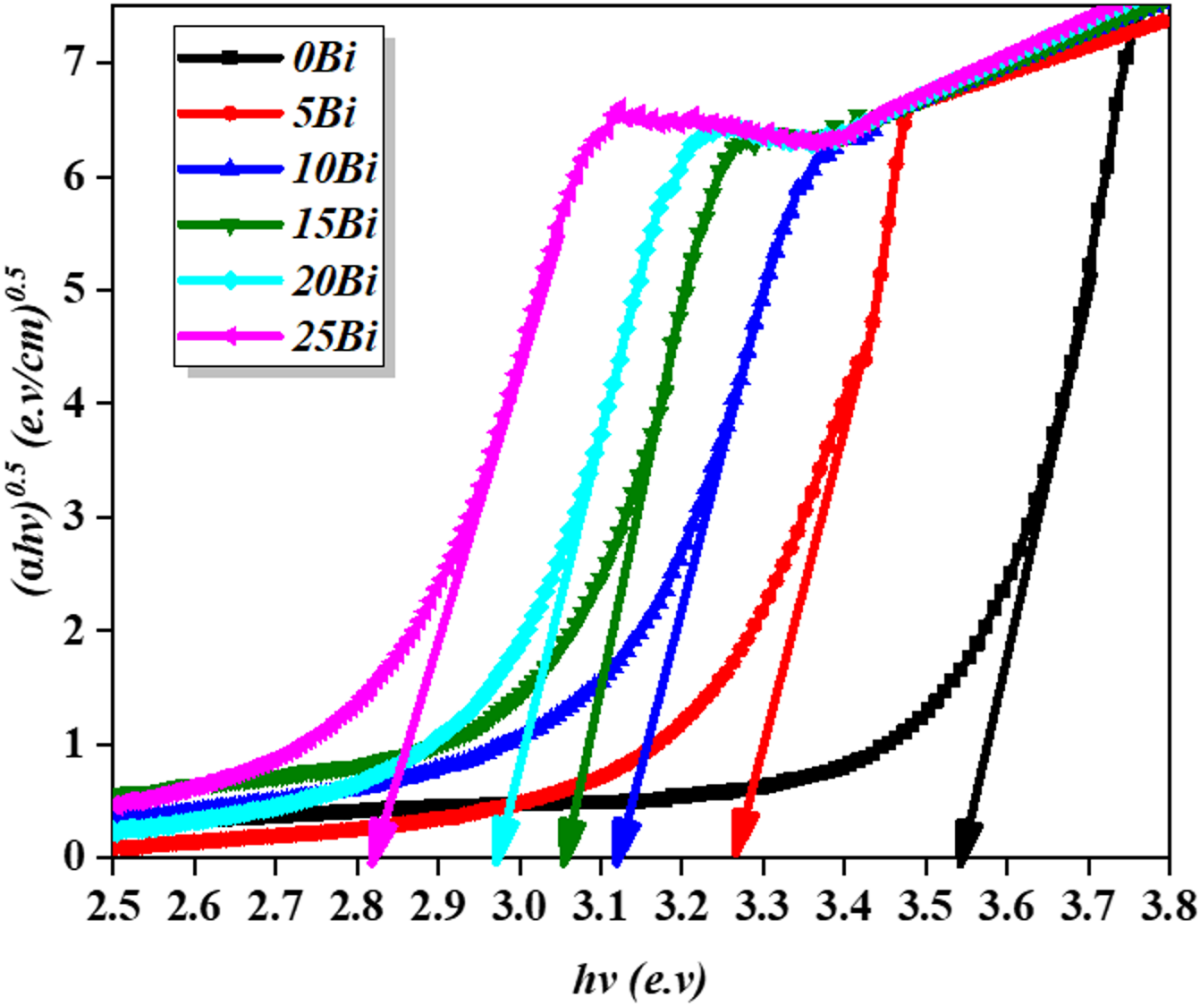
Tauc’s plot used to calculate the band gap energy across the glass samples.

The optical band gap ($\:{E}_{\mathrm{opt}}$) plays a critical role in determining the suitability of glass materials for various optical and electronic applications. It provides essential insight into the electronic structure and optical behavior of the studied system. A key step in this analysis is the examination of the redshift observed at the absorption edge, which reflects changes in the electronic structure with composition. Since the absorption edge is closely related to optical transitions in the UV region, accurately determining the optical band gap is essential. For indirect electronic transitions, the optical band gap can be evaluated using the well-known Mott–Davis relation^36^:

12 $$\:\alpha\:hv=B{(hv-{E}_{opt})}^{n}$$

where $\:\alpha\:$, $\:B$, and $\:h\nu\:$represent the absorption coefficient, a constant related to the glass structure, and the photon energy, respectively. The exponent $\:n$ depends on the type of electronic transition and takes values of 1/2 or 3/2 for direct transitions, and 2 or 3 for indirect transitions^4,35^. For amorphous materials, the permitted indirect band transition type is (*n* = 2). The values of $\:{E}_{opt}$ were determined by extrapolating the linear parts $\:{\left(\alpha\:hv\right)}^{\frac{1}{2}}$ versus to the x-axis at [ $\:\sqrt{\alpha\:hv}$ = 0] (which is additionally known as Tauc’s plot^37^. Figure 9 illustrates this relationship, and the corresponding values are summarized in Table 5. The optical band gap decreases significantly from 3.57 to 2.84 eV with increasing $\:{\mathrm{B}\mathrm{i}}_{2}{\mathrm{O}}_{3}$ content. This reduction is quantitatively correlated with the increase in NBO fraction from 13% to 26% and the rise in Urbach energy from 0.153 to 0.334 eV, indicating progressive structural disorder and the formation of localized states within the band gap. The formation of NBOs introduces defect levels that reduce the energy separation between the valence and conduction bands. This effect is further enhanced by the presence of Bi³⁺ ions, whose 6s² lone pair electrons increase electronic polarizability and promote the formation of additional states near the band edges. The weaker Bi–O bonds compared to B–O bonds facilitate the creation of mid-gap states, enabling electronic transitions at lower photon energies^4,35^. Additional contributions arise from the glass matrix components, including Ti³⁺/Ti⁴⁺ mixed valence states and Zr⁴⁺ and Ba²⁺ ions, which enhance structural disorder and NBO formation. However, these effects are secondary, while the dominant factor governing band gap narrowing is the $\:{\mathrm{B}\mathrm{i}}_{2}{\mathrm{O}}_{3}$-induced increase in NBO content and electronic polarizability^35,38^. Overall, the combined effects of Bi³⁺-induced NBO formation, mixed-valence Ti³⁺/Ti⁴⁺ states, Zr⁴⁺-induced structural disorder, and Ba²⁺/Ca²⁺-driven network modification lead to a systematic decrease in the optical band gap energy.

Urbach energy (ΔE) represents the width of the exponential tail of localized states near the band edge, which arises due to structural disorder, defects, and impurities in amorphous materials. It is a key parameter for evaluating the degree of disorder within the glass network. The Urbach energy was determined using the Urbach rule, expressed as^29,39^:

13 $$\:\alpha\:={\alpha\:}_{0\:}\mathrm{e}\mathrm{x}\mathrm{p}\left[\frac{hv}{{\Delta\:}\mathrm{E}}\right]$$

Where $\:{\alpha\:}_{0\:}$is a constant, the Urbach energy (ΔE) was obtained from the inverse of the slope of the linear region in the plot of $\:\mathrm{l}\mathrm{n}\left(\alpha\:\right)$versus $\:h\nu\:$ as follows:

14 $$\:\mathrm{ln}\left(\alpha\:\right)=\mathrm{ln}\left({\alpha\:}_{0}\right)+\:\frac{hv}{{\Delta\:}\mathrm{E}}$$

According to Table 5, ΔE increases from 0.153 to 0.334 eV with increasing $\:{\mathrm{B}\mathrm{i}}_{2}{\mathrm{O}}_{3}$ content, indicating an increase in structural disorder. This behavior is attributed to the formation of non-bridging oxygens (NBOs), which introduce localized states within the band gap and broaden the absorption edge. Moreover, the incorporation of Bi–O bonds, which are weaker and more flexible than B–O bonds, enhances electron–phonon interactions, thereby further tailing the absorption edge. In addition, the large ionic radius and asymmetric coordination of Bi^3+^ ions induce structural distortions within the glass network, increasing the density of localized states. Overall, these combined effects lead to the observed increase in Urbach energy, reflecting enhanced disorder and defect states in the glass structure^16,29^.

The linear refractive index ($\:{n}_{\circ\:}$) is closely related to the electronic polarizability of ions and the local field within the material. It is an important parameter for evaluating the suitability of glass materials in optical device applications. The refractive index can be estimated using the following empirical relation^39,40^:

15 $$\:{n}_{\circ\:}=\frac{\rho\:+10.4}{8.6}$$

The values of $\:{n}_{\circ\:}$ increase from 1.630 to 1.882 with increasing $\:{\mathrm{B}\mathrm{i}}_{2}{\mathrm{O}}_{3}$ content (see Table 4). This enhancement is mainly attributed to the higher electronic polarizability of Bi³⁺ ions (1.508 Å³) compared to B³⁺ ions (0.002 Å³). The incorporation of $\:{\mathrm{B}\mathrm{i}}_{2}{\mathrm{O}}_{3}$ promotes the breaking of bridging oxygen (BO) bonds and the formation of non-bridging oxygens (NBOs), which are more polarizable. The increase in NBO concentration enhances the overall polarizability of the glass network, thereby increasing the refractive index, in agreement with previous studies^15,39^.

The molar refractivity ($\:{R}_{m}$) is an important parameter that reflects a material’s overall polarizability on a molar basis. It is related to the refractive index and molar volume through the Lorentz–Lorenz equation, expressed as^41^:

16 $$\:{R}_{m}=\frac{{V}_{m}({{n}_{\circ\:}}^{2}-1)}{({{n}_{\circ\:}}^{2}+2)}\:$$

The molar electronic polarizability ($\:{\alpha\:}_{m}$) describes the response of the electron cloud to an external electric field and can be related to the molar refractivity using the following relation:

17 $$\:{\alpha\:}_{m}=\frac{3{R}_{m}}{4\pi\:{N}_{A}}$$

where $\:{N}_{A}$is Avogadro’s number.

According to the obtained results (Table 5), both $\:{R}_{m}$and $\:{\alpha\:}_{m}$ increase with increasing $\:{\mathrm{B}\mathrm{i}}_{2}{\mathrm{O}}_{3}$ content. This behavior is attributed to the high polarizability of Bi³⁺ ions, which enhances the distortion of the electron cloud within the glass network^15,22^. The observed increase in molar refractivity and electronic polarizability confirms the strong influence of bismuth incorporation on the optical properties of the glass system, making these materials promising candidates for optical and photonic applications.

The non-linear refractive index$\:\:{(n}_{2}$) refers to the component of the refractive index that relies on the intensity of the incident light. When nonlinear optical materials are exposed to high-intensity electromagnetic fields (such as laser light), the medium’s polarization responds nonlinearly, leading to a refractive index that depends on the intensity^41^. The non-linear refractive index is crucial in fields such as nonlinear optics, optical communications, and laser physics. Its calculation uses the Tichy-Ticha relation^41^:

18 $$\:{n}_{2}=\left[\frac{12\pi\:}{{n}_{\circ\:}}\right]{\chi\:}^{\left(3\right)}$$

Where $\:{\chi\:}^{\left(3\right)}$ refers to the 3rd order non-linear optical susceptibility. Furthermore, the representation of nonlinearity can be provided by the response of matter to light radiation that an expression for polarization of materials, which is expressed as an expansion of the power series in the electric field (E) based on this equation^41^:

19 $$\:\mathrm{P}\:={\chi\:}^{\left(1\right)}E+{\chi\:}^{\left(2\right)}{E}^{2}+{\chi\:}^{\left(3\right)}{E}^{3}+\dots\:$$

In which $\:{\chi\:}^{\left(1\right)}$, $\:{\chi\:}^{\left(2\right)}$and $\:{\chi\:}^{\left(3\right)}$ represent the 1st -order optical susceptibility, 2nd and 3rd - order non-linear optical susceptibility, in turn. E represents the incident light’s electric field. In addition, $\:{\chi\:}^{\left(1\right)}$ and $\:{\chi\:}^{\left(3\right)}$ could be assessed by following equations^41^:

20 $$\:{\chi\:}^{\left(1\right)}=\frac{{{n}_{\circ\:}}^{2}-1}{4\pi\:}$$

21 $$\:{\chi\:}^{\left(3\right)}=1.7\times\:{10}^{-10}{\left(\frac{{n}^{2}-1}{4\pi\:}\right)}^{4}$$

Table 5 presents the values of $\:{n}_{2}$, $\:{\chi\:}^{\left(1\right)}$ and $\:{\chi\:}^{\left(3\right)}$, which show a systematic increase with increasing $\:{\mathrm{B}\mathrm{i}}_{2}{\mathrm{O}}_{3}$ content. This enhancement can be explained based on the structural and electronic role of $\:{\mathrm{B}\mathrm{i}}^{3+}$ ions within the glass network. Bismuth oxide exhibits high electronic polarizability due to the presence of 6s² lone-pair electrons on $\:{\mathrm{B}\mathrm{i}}^{3+}$ ions, which can be easily distorted under an applied electric field. This leads to an increase in the overall electronic polarizability of the glass, resulting in higher values of the linear susceptibility $\:{\chi\:}^{\left(1\right)}$^42,43^. In addition, $\:{\mathrm{B}\mathrm{i}}_{2}{\mathrm{O}}_{3}$acts as a network modifier in the borate glass structure, breaking B–O–B linkages and generating non-bridging oxygens (NBOs). The increase in the NBO fraction (from 13% to 26%) introduces loosely bound electrons that are more readily polarized by an applied optical field, thereby enhancing local-field effects and strengthening the third-order nonlinear response. This structural evolution is quantitatively reflected in the significant increase in $\:{\chi\:}^{\left(3\right)}$ (from 5.16 to 19.6 × 10⁻¹⁴) and the nonlinear refractive index $\:{n}_{2}$ (from 1.19 to 4.07 × 10⁻¹²), indicating a strong correlation between NBO formation and nonlinear optical enhancement. Furthermore, Bi³⁺ ions, due to their large ionic radius and highly deformable electron cloud, contribute significantly to the overall electronic polarizability. This effect is reinforced by the increase in density (from 3.62 to 5.78 g/cm³), which enhances the local field strength and further promotes nonlinear polarization. In addition, the incorporation of $\:{\mathrm{B}\mathrm{i}}_{2}{\mathrm{O}}_{3}$ reduces the optical band gap (3.57–2.84 eV), facilitating nonlinear optical transitions. Therefore, the combined effects of increased NBO content, higher density, and enhanced polarizability lead to the observed improvement in $\:{\chi\:}^{\left(3\right)}$ and $\:{n}_{2}$^44^. Overall, the observed enhancement in $\:{n}_{2}$, $\:{\chi\:}^{\left(1\right)}$, and $\:{\chi\:}^{\left(3\right)}$can be attributed to: (i) the high polarizability of Bi³⁺ ions, (ii) the formation of non-bridging oxygens, (iii) increased structural asymmetry and local field effects, and (iv) band gap reduction promoting nonlinear optical transitions. These results demonstrate the strong potential of the studied glasses for photonic applications, including wavelength conversion, ultrafast optical switching, and nonlinear waveguiding^45^.

**Table 5:** The wavelength of optical transitions of $\:{\mathrm{T}\mathrm{i}}^{3+}$ ions, ligand field strength (10Dq), optical band gap energy ($\:{E}_{opt}$), Urbach’s energy (ΔE), linear refractive index ($\:{n}_{\circ\:}$), molar refractivity ($\:{R}_{m}$), molar- electronic polarizability ($\:{\alpha\:}_{m}$), non-linear refractive index ($\:{n}_{2}$), linear optical susceptibility ($\:{\chi\:}^{\left(1\right)}$) and non-linear optical susceptibility $\:{\chi\:}^{\left(3\right)}$ of all glass samples.

Sample (mol%)	$\:{}^{2}{\boldsymbol{B}}_{2\boldsymbol{g}}\to\:{}^{2}{\boldsymbol{B}}_{1\boldsymbol{g}}$ (nm)	$\:{}^{2}{\mathbf{B}}_{2\mathbf{g}}\to\:$ $\:{}^{2}{\mathbf{A}}_{1\mathbf{g}}$ (nm)	10Dq ($\:{\boldsymbol{c}\boldsymbol{m}}^{-1}$) $\:\pm\:$2.12	$\:{\boldsymbol{E}}_{\boldsymbol{o}\boldsymbol{p}\boldsymbol{t}}$ (eV)	ΔE (eV)	$\:{\boldsymbol{n}}_{\circ\:}$	$\:{\boldsymbol{R}}_{\boldsymbol{m}}$ ($\:{\mathbf{c}\mathbf{m}}^{3}/\mathbf{m}\mathbf{o}\mathbf{l})$	$\:{\boldsymbol{\alpha\:}}_{\boldsymbol{m}}$ (×$\:{10}^{-24}$ $\:{\mathbf{c}\mathbf{m}}^{3}$)	$\:{\boldsymbol{n}}_{2}$ (×$\:{10}^{-12}$)	$\:{\boldsymbol{\chi\:}}^{\left(1\right)}$	$\:{\boldsymbol{\chi\:}}^{\left(3\right)}$ (×$\:{10}^{-14}$)
0 Bi	501	632	1994	3.61	0.153	1.63	9.72	3.85	1.19	0.13	5.16
5 Bi	500	626	1998	3.33	0.191	1.67	11	4.39	1.71	0.14	7.67
10 Bi	499	625	2002	3.12	0.204	1.73	12.2	4.87	2.42	0.16	11.1
15 Bi	498	624	2006	3.05	0.261	1.77	13.6	5.40	3.49	0.17	16.6
20 Bi	497	623	2010	2.99	0.312	1.82	14.7	5.85	4.07	0.18	19.6
25 Bi	496	619	2012	2.83	0.334	1.88	15.6	6.22	5.71	0.20	28.5

### Gamma-ray shielding characteristics

Phy-X/PSD software was employed to estimate the gamma-ray attenuation characteristics of the prepared glass samples over a photon energy range of 15 keV to 15 MeV. This software is widely used for reliable evaluation of radiation shielding parameters based on the material composition and density. The shielding performance of the investigated glasses was analyzed in terms of key parameters, including the mass attenuation coefficient (MAC), linear attenuation coefficient (LAC), mean free path (MFP), half-value layer (HVL), and effective atomic number ($\:{Z}_{\mathrm{e}\mathrm{f}\mathrm{f}}$). The calculations were performed using the XCOM database, ensuring accurate estimates of photon interaction parameters.

#### Linear attenuation coefficient (LAC)

**Fig. 10:**
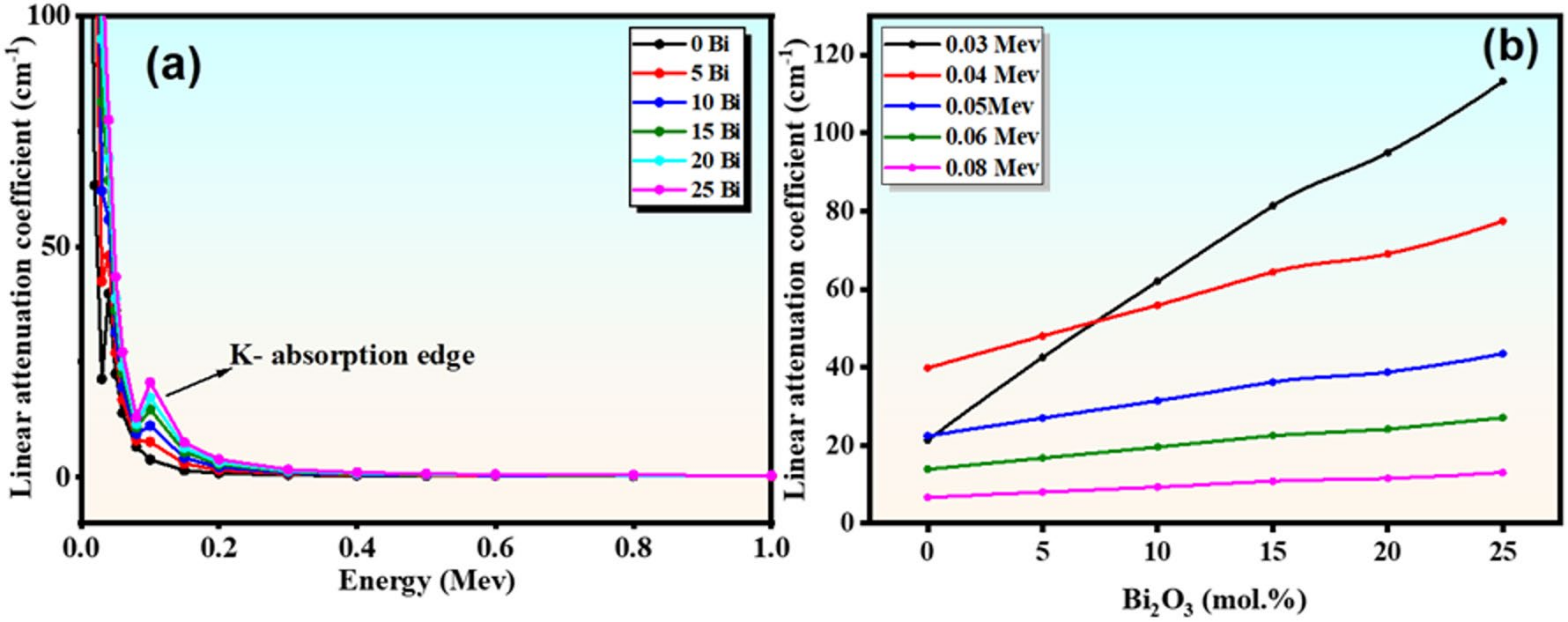
(**a**) Linear attenuation coefficient (LAC) concerning photon energies across glass samples; (**b**) Various values of linear attenuation coefficient with Bi content at varying photon energies.

The linear attenuation coefficient (LAC) of the prepared $\:{\mathrm{B}\mathrm{i}}_{2}{\mathrm{O}}_{3}$–BCZT borate glasses was evaluated over the photon energy range of 0.015–15 MeV. As a density-dependent parameter, LAC serves as a key indicator of a material’s ability to attenuate gamma radiation^46,47^. Figure 10(a) illustrates the variation of LAC with photon energy for the investigated glass series (S1–S6). The results show that LAC values increase systematically with increasing $\:{\mathrm{B}\mathrm{i}}_{2}{\mathrm{O}}_{3}$ content across all energy ranges. At low photon energy (0.015 MeV), LAC values increase significantly from 122.128 to 451.621 cm⁻¹, while at higher energies they decrease to 1.504–7.542 cm⁻¹ (0.15 MeV), 0.175–0.294 cm⁻¹ (1.5 MeV), and 0.114–0.264 cm⁻¹ (15 MeV) for samples containing 0–25 mol% $\:{\mathrm{B}\mathrm{i}}_{2}{\mathrm{O}}_{3}$. This behaviour is governed by photon interaction mechanisms. At low energies (0.015–0.3 MeV), photoelectric absorption dominates, resulting in high LAC values. In the intermediate energy region (0.3–5 MeV), Compton scattering becomes dominant, leading to a gradual decrease. At higher energies (> 5 MeV), pair production contributes, causing a slight variation.

Figure 10(b) further demonstrates the strong influence of $\:{\mathrm{B}\mathrm{i}}_{2}{\mathrm{O}}_{3}$ content, where LAC increases markedly with increasing bismuth concentration. For instance, at 0.03 MeV, LAC increases from 21.26 to 113.204 cm⁻¹, while at 0.08 MeV, it rises from 6.609 to 12.955 cm⁻¹ as $\:{\mathrm{B}\mathrm{i}}_{2}{\mathrm{O}}_{3}$ content increases from 0 to 25 mol%. This enhancement is directly related to the increase in density and the presence of high-Z Bi ions, which increase the probability of photon interactions^47^. Consequently, glasses with higher $\:{\mathrm{B}\mathrm{i}}_{2}{\mathrm{O}}_{3}$ content exhibits superior shielding performance across low, intermediate, and high photon energy regions.

#### Mass attenuation coefficient

The mass attenuation coefficient (MAC) is a fundamental parameter for evaluating the radiation shielding performance of glass materials. It describes the probability of gamma-ray interaction per unit mass and is therefore independent of sample geometry, making it highly suitable for comparative evaluation of different glass compositions^15^. MAC was calculated using Phy-X/PSD over the energy range 0.015–15 MeV.

**Fig. 11:**
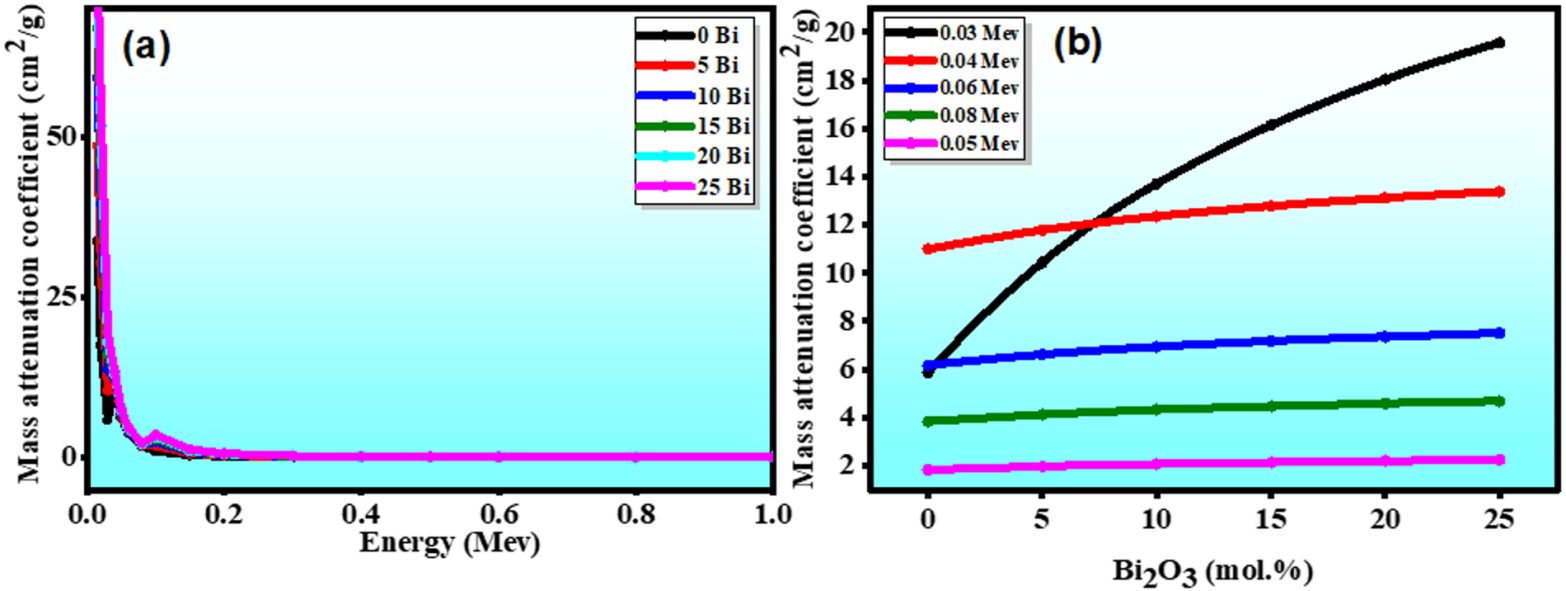
(**a**) MAC for photon energies across the glass samples; (**b**) Various values of MAC with Bi content at varying photon energies.

Figure 11(a) shows how the energy of the incident ionizing radiation affects the MAC behaviors of all glass samples, which exhibit distinct trends in the various energy zones. The obtained results revealed that the highest MAC values were at lower energies (15 keV), ranging from 33.718 to 78.027 cm²/g, indicating strong photoelectric absorption. MAC values decrease sharply with increasing energy up to ~ 1 MeV, with the exception of a sudden 0.1 MeV increase. The dominant Photoelectric Effect (PE) at low energies, from 0.015 to 0.3 MeV, is responsible for this rapid drop in MAC values. Furthermore, the K-edge of the Bi element in the glass matrix results in a sharp peak at about 0.1 MeV. This effect appears when the energy of the incoming photons becomes just higher than the binding energy of the innermost electrons (K-shell). At this point, the photons can easily eject these tightly bound electrons, leading to a sharp rise in photon absorption by the material^48^. The dominant Compton Scattering (CS) was followed by a minor variation in the MAC values for all glass samples across input photon energies from 0.3 to 5 MeV. The behavior becomes nearly constant as the photon energy increases up to 5 MeV, suggesting a pair-creation process that is dependent on log E^47^. Importantly, across all energies, the MAC values increased consistently with $\:{\mathrm{B}\mathrm{i}}_{2}{\mathrm{O}}_{3}$ substitution as shown in Fig. 11(b). It is obvious that 25 $\:{\mathrm{B}\mathrm{i}}_{2}{\mathrm{O}}_{3}$ has the highest and greatest MAC values among all the created glasses, whilst the 0 $\:{\mathrm{B}\mathrm{i}}_{2}{\mathrm{O}}_{3}$ sample has the lowest MAC. Interestingly, as the $\:{\mathrm{B}\mathrm{i}}_{2}{\mathrm{O}}_{3}$ percentage rises, the MAC value at a given energy increases, indicating an improvement in the attenuation characteristics of the produced glasses. This suggests that samples 20 and 25 $\:{\mathrm{B}\mathrm{i}}_{2}{\mathrm{O}}_{3}$ are better suited for ionizing radiation protection fields^48^.

#### Half value layer (HVL)

**Fig. 12:**
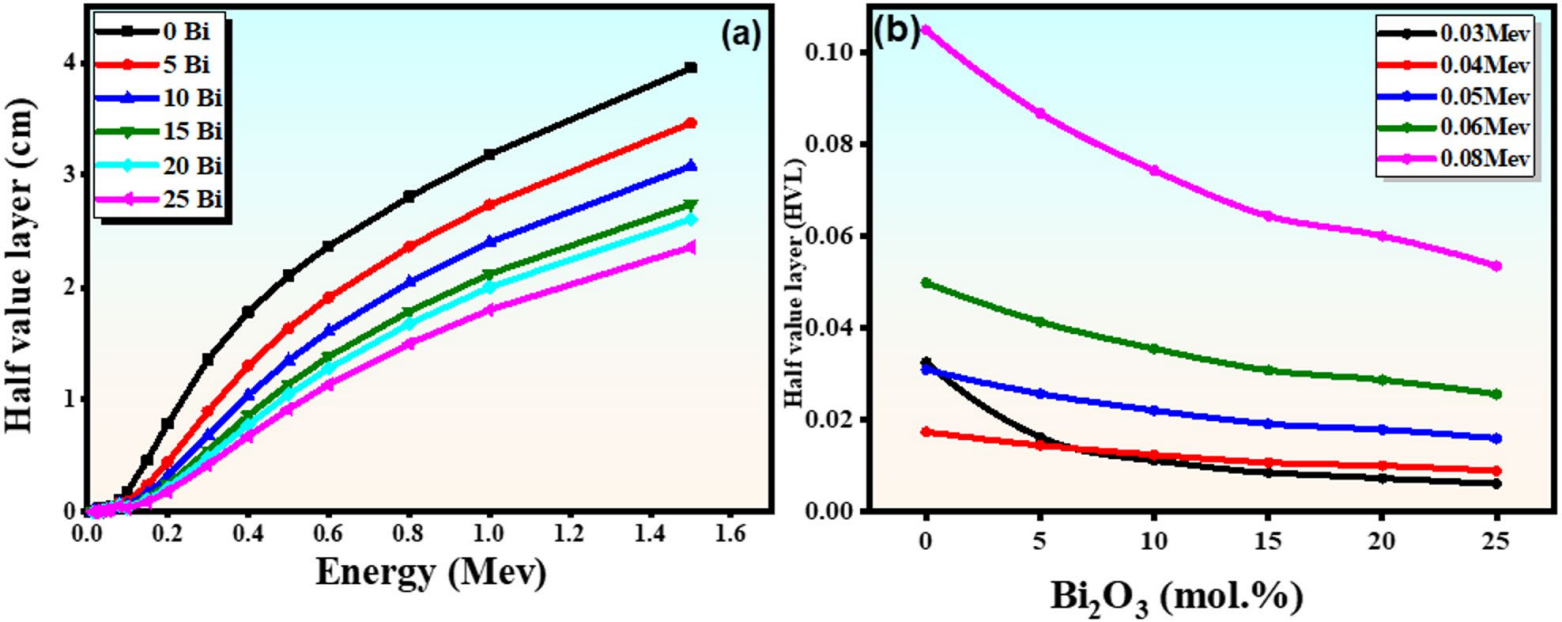
(**a**) Half-value layer represented as one of the functions of photon energy across the glass samples; (**b**) half-value layer as a function of $\:{\mathrm{B}\mathrm{i}}_{2}{\mathrm{O}}_{3}$ content concerning the studied glass samples.

The half-value layer (HVL) is a key shielding parameter that represents the thickness of a material required to reduce the intensity of incident gamma radiation to half of its initial value. It is inversely related to the linear attenuation coefficient, as given by (HVL = ln2 /µ$\:\mathrm{)}$, and therefore provides a direct measure of a material’s attenuation efficiency. A lower HVL value indicates superior shielding performance^46^. As shown in Fig. 12(a), the HVL decreases with increasing $\:{\mathrm{B}\mathrm{i}}_{2}{\mathrm{O}}_{3}$ content, indicating enhanced gamma-ray attenuation. This improvement can be attributed to the increase in both the density and the effective atomic number of the glass system upon incorporation of $\:{\mathrm{B}\mathrm{i}}_{2}{\mathrm{O}}_{3}$. The presence of high-Z Bi³⁺ ions enhances photon–matter interaction probabilities, particularly through the photoelectric absorption mechanism, which is strongly dependent on atomic number. Consequently, an increase in the mass attenuation coefficient (µ/ρ) results in a higher linear attenuation coefficient (µ), leading to a significant reduction in HVL. At 0.6 MeV, the HVL values decrease from 2.369 cm (0 mol% $\:{\mathrm{B}\mathrm{i}}_{2}{\mathrm{O}}_{3}$) to 1.133 cm (25 mol% $\:{\mathrm{B}\mathrm{i}}_{2}{\mathrm{O}}_{3}$), confirming the substantial improvement in shielding efficiency with increasing bismuth content. Furthermore, the HVL values of the studied glasses are lower than those of common shielding materials such as commercial glass (4.73 cm), concrete (3.65 cm), ilmenite (2.63 cm), and serpentine (4.07 cm), demonstrating the superior shielding performance of the present glass system.

Figure 12(b) shows that HVL values are lowest at low photon energies (e.g., 0.03 MeV), where the photoelectric effect dominates, resulting in strong attenuation. In contrast, the HVL values increase at intermediate energies (around 0.8 MeV), where Compton scattering is the dominant interaction mechanism^49^. The observed enhancement is more pronounced at lower photon energies, where the photoelectric effect dominates due to its strong dependence on atomic number (Z⁴–Z⁵).

#### Mean free path (MFP)

**Fig. 13:**
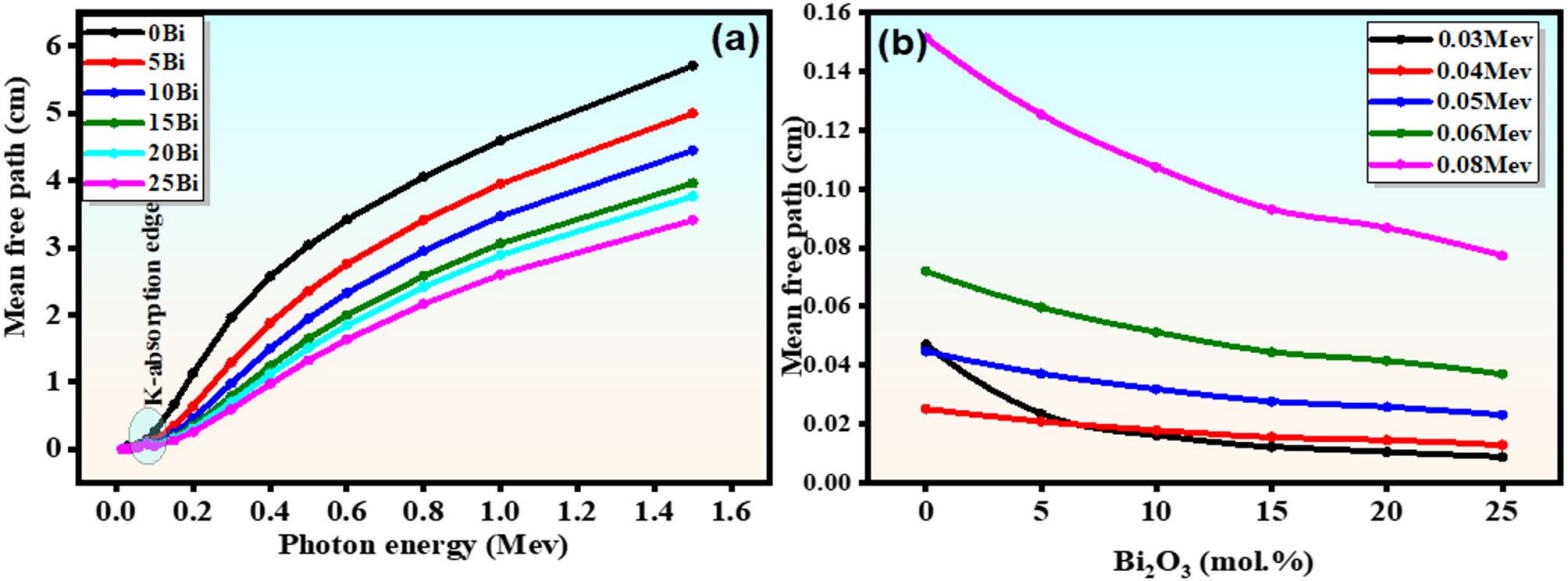
(**a**) Mean free path (MFP) represented as one of the functions of photon energies across the glass samples; (**b**) Various values of MFP with Bi at several photon energies.

The mean free path (MFP) is a key shielding parameter that represents the average distance a photon travels in a material before interaction and is inversely related to the linear attenuation coefficient (MFP = 1/µ)^47^. Therefore, lower MFP values indicate a higher probability of photon interaction and better shielding efficiency. As shown in Fig. 13(a), MFP increases with photon energy due to the reduction in photon–matter interaction cross-section at higher energies. Distinct features observed at low energies correspond to the K-edge absorption of Bi, confirming the strong influence of high-Z elements on photon attenuation behavior.

At a fixed photon energy (Fig. 13(b)), MFP decreases systematically with increasing $\:{\mathrm{B}\mathrm{i}}_{2}{\mathrm{O}}_{3}$ content, which is quantitatively consistent with the increase in density (3.62–5.78 g/cm³) and effective atomic number. This enhancement increases the interaction probability and reduces the photon penetration depth. These results indicate that $\:{\mathrm{B}\mathrm{i}}_{2}{\mathrm{O}}_{3}$ incorporation significantly improves shielding performance, allowing thinner glass thicknesses to achieve effective attenuation. However, it should be noted that the MFP values are derived from theoretical calculations (Phy-X/XCOM), and therefore represent comparative trends, while experimental validation may be required for precise absolute evaluation^15^. These trends are consistent with the HVL results and are further explained by the variation of $\:{\mathrm{Z}}_{\mathrm{e}\mathrm{f}\mathrm{f}}$ in the following section.

#### Effective atomic number ($\:{\mathbf{Z}}_{\mathbf{e}\mathbf{f}\mathbf{f}}$)

The effective atomic number ($\:{\mathrm{Z}}_{\mathrm{e}\mathrm{f}\mathrm{f}}$) is a crucial shielding parameter that reflects the composite material’s overall atomic number and its effectiveness in photon attenuation. It was evaluated using the Phy-X/PSD software based on the elemental composition and photon energy. The calculations rely on the XCOM database, which includes photon interaction cross-sections for photoelectric absorption, Compton scattering, and pair production, ensuring accurate estimates of shielding parameters. Unlike the atomic number of a single element, $\:{\mathrm{Z}}_{\mathrm{e}\mathrm{f}\mathrm{f}}$ represents a weighted average that accounts for both the elemental composition and energy-dependent photon-interaction mechanisms. A higher $\:{\mathrm{Z}}_{\mathrm{e}\mathrm{f}\mathrm{f}}$ value indicates a greater probability of photon interaction and, consequently, improved gamma-ray shielding performance.

**Fig. 14:**
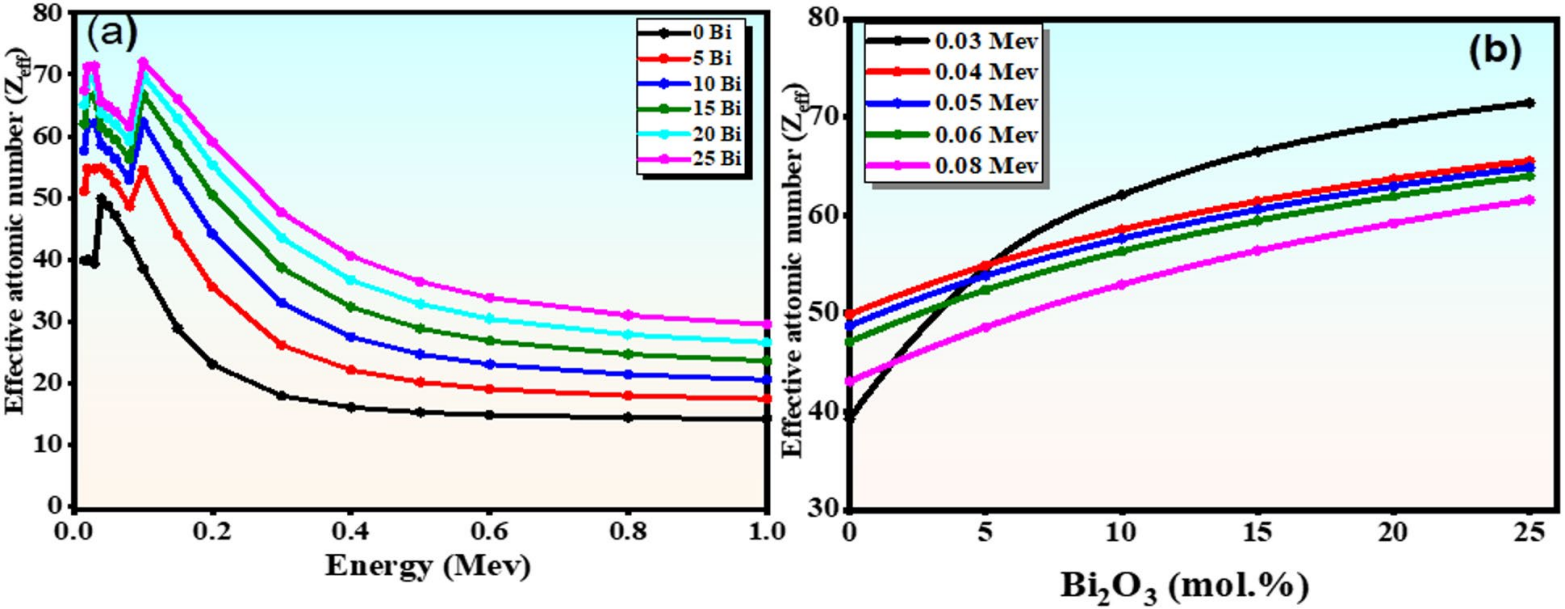
(**a**) Effective atomic number ($\:{\mathrm{Z}}_{\mathrm{e}\mathrm{f}\mathrm{f}}$) presented as a function of photon energies across the glass samples; (**b**) Various values of $\:{\mathrm{Z}}_{\mathrm{e}\mathrm{f}\mathrm{f}}$ and the incorporation of $\:{\mathrm{B}\mathrm{i}}_{2}{\mathrm{O}}_{3}$ at several photon energies.

As shown in Fig. 14(a), $\:{\mathrm{Z}}_{\mathrm{e}\mathrm{f}\mathrm{f}}$ decreases with increasing photon energy due to the transition from photoelectric absorption at low energies to Compton scattering at intermediate energies. At low photon energies (0.015–0.15 MeV), $\:{\mathrm{Z}}_{\mathrm{e}\mathrm{f}\mathrm{f}}$ values decrease (e.g., from 39.81 to 67.4 to 28.85–65.97), reflecting the reduced dominance of photoelectric interactions. The observed fluctuations correspond to the K-edge absorption of constituent elements, particularly Bi, confirming its strong influence on photon interaction behavior^48^. At the intermediate energy range (0.15–0.5 MeV), $\:{\mathrm{Z}}_{\mathrm{e}\mathrm{f}\mathrm{f}}$ exhibits a steeper decline, which is associated with the transition from electron-dominated interactions to a regime where photon interactions with the nuclei of higher-Z elements, such as $\:{\mathrm{B}\mathrm{i}}^{3+}$ ions, become more significant. This behavior highlights the composite nature of the glass and confirms that $\:{\mathrm{B}\mathrm{i}}_{2}{\mathrm{O}}_{3}$ incorporation enhances the contribution of heavy-element interactions, thereby improving photon attenuation efficiency, particularly in the intermediate- and high-energy regions. Importantly, $\:{\mathrm{Z}}_{\mathrm{e}\mathrm{f}\mathrm{f}}$ increased systematically with $\:{\mathrm{B}\mathrm{i}}_{2}{\mathrm{O}}_{3}$ incorporation in Fig. 14(b), which is directly attributed to the high atomic number of bismuth (Z = 83). For example, at low photon energies (< 0.1 MeV), glasses with 25 mol% $\:{\mathrm{B}\mathrm{i}}_{2}{\mathrm{O}}_{3}$ showed markedly higher $\:{\mathrm{Z}}_{\mathrm{e}\mathrm{f}\mathrm{f}}$ compared to Bi-free compositions, confirming the strong role of Bi in enhancing photon–matter interactions^50^.

This behavior is consistent with the reduction in HVL and MFP values, confirming that $\:{\mathrm{B}\mathrm{i}}_{2}{\mathrm{O}}_{3}$ incorporation enhances photon interaction probability and improves shielding efficiency across a wide energy range.

It should be noted that $\:{\mathrm{Z}}_{\mathrm{e}\mathrm{f}\mathrm{f}}$ values are derived from standard theoretical models and interpolation methods (XCOM/Phy-X). While these approaches provide reliable estimates within the studied energy range, their accuracy may be limited at very low or very high photon energies due to the complex nature of photon interaction mechanisms. Therefore, the obtained values should be considered as approximate indicators of shielding performance rather than absolute quantities. Overall, these results demonstrate that $\:{\mathrm{B}\mathrm{i}}_{2}{\mathrm{O}}_{3}$-rich borate glasses exhibit enhanced effective atomic numbers, confirming their potential as efficient and compact gamma-ray shielding materials.

To further evaluate the performance of the present glasses, a comparison with recent literature (2025–2026) is summarized in Table 6.

**Table 6:** Comparison of the present work with recent borate-based glasses (2025–2026).

Property	Present work	Serag et al. (2026)	Helmy et al. (2025)	Iliyasu et al. (2025)
Structure	Amorphous	Amorphous	Amorphous	Amorphous
Glass system	$\:{\mathrm{B}\mathrm{i}}_{2}{\mathrm{O}}_{3}$–$\:{\mathrm{B}}_{2}{\mathrm{O}}_{3}$–BCZT	$\:{\mathrm{B}}_{2}{\mathrm{O}}_{3}$–$\:{\mathrm{B}\mathrm{i}}_{2}{\mathrm{O}}_{3}$–ZnO–BaO	Bismuth doped lithium borosilicate glass	$\:{\mathrm{B}}_{2}{\mathrm{O}}_{3}-\mathrm{Z}\mathrm{n}\mathrm{O}-\mathrm{P}\mathrm{b}$_3_
Density	3.62–5.78 g/cm³	Moderate ↑	2.31–4.59 g/cm³	4.17–5.26 g/cm³
BO₃ → BO₄	60% to 87%	Not Reported	0.66%-75%	Not Reported
Band gap (eV)	3.61–2.99 eV	4.89–4.18 eV	3.44–2.39 eV	2.99–2.8 eV
Urbach energy	0.153–0.334 eV	0.86–0.94 eV	0.2164–0.488 eV	0.38–0.46 eV
NLO (χ³, n₂)	High enhancement	Enhanced	Limited	Not reported clearly
Shielding (MAC)	33.718–78.027 $\:{\mathrm{c}\mathrm{m}}^{2}$/g at 15 keV	Not main focus	Reaching 1.106 × 10⁻¹ cm²/g at 0.662 MeV	0.95–1.357 $\:{\mathrm{c}\mathrm{m}}^{2}$/g at 0.15 MeV
HVL	2.36 → 1.11	—	Reduced	Not reported
Key feature	Multifunctional	Optical/NLO	Optical + shielding	Structural + shielding

## Conclusion

- This study successfully addresses the research gap related to the lack of integrated understanding of the multifunctional role of $\:{\mathrm{B}\mathrm{i}}_{2}{\mathrm{O}}_{3}$ in borate–BCZT glass systems, particularly in correlating structural modifications with optical, nonlinear, and radiation shielding properties.
- The investigated glasses were successfully fabricated using B₂O₃ as a glass former at relatively low concentrations down to 5 mol%, while preserving the amorphous structure and key physical properties of the glass system. To the best of our knowledge, achieving stable borate–BCZT glasses at such low $\:{\mathrm{B}}_{2}{\mathrm{O}}_{3}$ content without compromising structural integrity and multifunctional performance represents a novel contribution in this field.
- The incorporation of $\:{\mathrm{B}\mathrm{i}}_{2}{\mathrm{O}}_{3}$ preserved the amorphous nature of the glasses while significantly increasing the density (3.622–5.788 g/cm³). Structural analysis revealed network modification through Bi–O bond formation and a progressive transformation from $\:{\mathrm{B}\mathrm{O}}_{3}$ to $\:{\mathrm{B}\mathrm{O}}_{4}$ units, accompanied by an increase in non-bridging oxygen content (13.42–26.25%), confirming effective restructuring of the glass network.
- Optical analysis showed a reduction in band gap from 3.61 to 2.99 eV and an increase in Urbach energy from 0.153 to 0.312 eV, indicating increased structural disorder.
- Nonlinear optical properties were significantly enhanced, with $\:{n}_{2}$ increasing from 1.19 to 4.07 × 10⁻¹² and $\:{\chi\:}^{\left(3\right)}$ from 5.16 to 19.6 × 10⁻¹⁴ due to increased polarizability and NBO formation.
- Gamma-ray shielding performance improved, with high MAC values at low energy (15 keV) and a decrease in HVL from 2.369 to 1.113 cm at 0.6 MeV, confirming superior attenuation efficiency.

In summary, the obtained results demonstrate that controlled incorporation of $\:{\mathrm{B}\mathrm{i}}_{2}{\mathrm{O}}_{3}$, provides an effective strategy to tailor the structural and functional properties of borate-based glasses for multifunctional applications in optics and radiation protection. Furthermore, the successful formation of stable borate–BCZT glasses using $\:{\mathrm{B}}_{2}{\mathrm{O}}_{3}$ as a glass former at low concentrations down to 5 mol%, without compromising the amorphous structure or key properties, represents a novel contribution in this field.

## Data Availability

Data will be made available on request.
